# Structural Analyses of a Dominant Cryptosporidium parvum Epitope Presented by H-2K^b^ Offer New Options To Combat Cryptosporidiosis

**DOI:** 10.1128/mbio.02666-22

**Published:** 2023-01-05

**Authors:** Yongli Wang, Minghui Gao, Xiaoying Li, Wenshuai Zhu, Mengchun Zhao, Jiayue Li, Xiaomeng Liu, Letian Cao, Songrui Li, Sumei Zhang, Longxian Zhang, Shuhua Fan

**Affiliations:** a College of Life Science and Agronomy, Zhoukou Normal University, Zhoukou, People’s Republic of China; b College of Veterinary Medicine, Henan Agricultural University, Zhengzhou, People’s Republic of China; c Institute of Neuroscience and Translational Medicine, Zhoukou Normal University, Zhoukou, People’s Republic of China; d State Key Laboratory of Agricultural Microbiology, College of Veterinary Medicine, Huazhong Agricultural University, Wuhan, People’s Republic of China; Tufts Medical Center; Harvard T. H. Chan School of Public Health

**Keywords:** *Cryptosporidium parvum*, MHC I, T-cell epitope, cell-mediated immunity

## Abstract

Cryptosporidium parvum has gained much attention as a major cause of diarrhea in the world, particularly in those with compromised immune systems. The data currently available on how the immune system recognizes C. parvum are growing rapidly, but we lack data on the interactions among host major histocompatibility complex (MHC) diversity and parasitic T-cell epitopes. To identify antigenic epitopes in a murine model, we performed systematic profiling of H-2K^b^-restricted peptides by screening the dominant *Cryptosporidium* antigens. The results revealed that the glycoprotein-derived epitope Gp40/15-SVF9 induced an immunodominant response in C. parvum-recovered C57BL/6 mice, and injection of the cytotoxic-T-lymphocyte (CTL) peptide with the adjuvant activated peptide-specific CD8^+^ T cells. Notably, the SVF9 epitope was highly conserved across Cryptosporidium hominis, C. parvum, and many other *Cryptosporidium* species. SVF9 also formed stable peptide-MHC class I (MHC I) complexes with HLA-A*0201, suggesting cross-reactivity between H-2K^b^ and human MHC I specificities. Crystal structure analyses revealed that the interactions of peptide-MHC surface residues of H-2K^b^ and HLA-A*0201 are highly conserved. The hydrogen bonds of H-2K^b^–SVF9 are similar to those of a dominant epitope presented by HLA-A*0201, which can be recognized by a public human T-cell receptor (TCR). Notably, we found double conformations in position 4 (P4), 5 (P5) of the SVF9 peptide, which showed high flexibility, and multiple peptide conformations generated more molecular surfaces that can potentially be recognized by TCRs. Our findings demonstrate that an immunodominant C. parvum epitope and its homologs from different *Cryptosporidium* species and subtypes can benefit vaccine development to combat cryptosporidiosis.

## INTRODUCTION

*Cryptosporidium*, an opportunistic intracellular parasite, is a major cause of diarrheal illness around the world. Cryptosporidium parvum is among the few *Cryptosporidium* species with a broad host range and is the most important zoonotic species (1). It can infect humans, domestic animals, and wild animals and can be life-threatening in immunocompromised hosts such as individuals with AIDS (2–4). Approximately 90% of human infections are caused predominantly by Cryptosporidium hominis and C. parvum, whose transmission routes are primarily waterborne, foodborne, or by contact with infected people or animals (1, 5). This massive impact on public health is exacerbated by the lack of tools to manage the disease; no efficacious vaccine is available to prevent the infection, the current treatment for *Cryptosporidium* is considered ineffective in immunocompromised individuals, and new, more potent medicines are still under development (6–9).

The increased severity and duration of illness observed in immunocompromised individuals suggest that resistance to and resolution of C. parvum infection require the activation of the host immune system, and vaccination may prevent the disease (10–13). Although the crucial roles of T-cell-mediated immunity to *Cryptosporidium* in the host immune system have been elaborated, information regarding the precise targets of these cells remains remarkably incomplete (14–17). The interactions among host major histocompatibility complex (MHC) diversity and parasite epitopes have been ignored, and few studies have examined the association between mouse MHC I (MHC class I) alleles and C. parvum proteins.

Mouse MHC I is also called histocompatibility system 2 (H-2), which is located on chromosome 17, and there are 3 classically expressed MHC I genes, H-2K, H-2D, and H-2L. Previous work has shown that mouse MHC I is involved in the regulation of parasitic infection in mice and is closely associated with disease resistance and susceptibility (18, 19). MHC I and CD8^+^ T-cell epitopes interact in the peptide-binding groove of MHC class I molecules and form the peptide-MHC I (pMHC I) complex (20). The pMHC complexes are translocated to the cell surface and recognized by T lymphocytes with specific T-cell receptors (TCRs), resulting in specific cellular immune responses and pathogen elimination from the host (21). However, not all of the peptide-MHC I complexes that are recognizable are equal; rather, they elicit a hierarchy of specific T cells (21, 22). The peptides that elicit the most abundant cognate T-cell populations are called immunodominant epitopes. Knowledge of the mechanisms that enhance immunogenicity and determine the immunodominance hierarchy is central to being able to design efficacious vaccines (22). The CD8^+^ T-cell epitopes in other intracellular parasites such as *Plasmodium*, Trypanosoma cruzi, Toxoplasma gondii, and Theileria parva linked with human and mouse MHC I molecules have been identified by MHC ligand assays and T-cell assays, such as interferon gamma (IFN-γ) release and pathogen burdens after challenge (21–26). However, our understanding of the T-cell epitopes for *Cryptosporidium* and MHC I molecules remains limited.

The invasion and infection processes of C. parvum rely on the numerous surface and/or apical complex proteins involved in the attachment to or penetration of host cells (27, 28). These proteins include many surface antigens (Ags) such as circumsporozoite-like (CSL), glycoprotein 900 (Gp900), *Cryptosporidium* protein 23 (Cp23), Gp40/15 (also referred to as Gp60), and Cp15. Previous studies have shown that vaccination with a short synthetic peptide in Freund’s adjuvant results in protective antiviral, antitumor, and antiparasitic T-cell immunity (29–31). Therefore, we wondered whether vaccination with the dominant *Cryptosporidium* antigen epitopes could induce antigen-specific T-cell responses and attenuate the severity of C. parvum infection.

Here, we screened the main surface proteins and found that H-2K^b^ might bind with more C. parvum epitopes than other mouse MHC I alleles. To further identify H-2K^b^-restricted cytotoxic-T-lymphocyte (CTL) epitopes and T-cell responses in the C57BL/6 mouse model (which expresses the H-2^b^ haplotype), fast protein liquid chromatography (FPLC), an enzyme-linked immunosorbent spot assay (ELISpot), and intracellular cytokine staining (ICS) were employed with *in vitro* and *in vivo* assays. In addition, using the structural landscape of H-2K^b^ bound to the Gp40/15-SVF9 peptide (SVFAIFAAL), we delineated the molecular basis for immunodominant C. parvum Gp40/15-derived T-cell epitope cross-reactivity toward H-2K^b^ and human HLA-A*0201. Structural and biochemical analyses of protective epitopes from *Cryptosporidium* proteins should not only underscore the role of Ag-specific T-cell responses in protective immunity against C. parvum infection but also advance our understanding of T-cell immune responses toward *Cryptosporidium* and, by extension, assist in vaccine development against cryptosporidiosis.

## RESULTS

### Epitope prediction for novel C. parvum proteins.

A computer-based program was used to identify the potential mouse H-2 allele-specific peptides from C. parvum (32). The results of *in silico* prediction indicated that H-2K^b^ might bind to more C. parvum epitopes than other mouse MHC I alleles. Interestingly, H-2K^b^-restricted epitopes in C57BL/6 mice were found to partly overlap peptides of human HLA-A*0201, one of the most prevalent HLAs in the global population (33). According to data from the Allele Frequency Net Database (http://www.allelefrequencies.net) (34), the HLA-A*0201 allele is widely distributed in South America, North America, Europe, Africa, and other countries and regions. The geographic distributions of HLA-A*0201 and the worldwide distribution of C. parvum outbreaks and morbidity are shown in Table S1 in the supplemental material. Next, we screened the main surface protein sequences, including Gp40/15, Cp23, Cp15, Gp900, and CSL, from C. parvum using NetMHCpan4.0, from which the algorithm generated peptide predictions for H-2K^b^ and HLA-A*0201 alleles. Altogether, 202 8- to 11-mer peptides were predicted to fit the H-2K^b^ or HLA-A*0201 motifs, including 107 H-2K^b^-restricted peptides and 110 HLA-A*0201-restricted peptides. They had theoretical 50% inhibitory concentration (IC_50_) values ranging from 2.9 to 1179.35 nM, with the levels of peptide binding to the MHC ranked as SB (strongly binding peptides, which is defined as a percentage rank of affinity (%rank) of less than 0.5) (%Rank < 0.500) or WB (weakly binding peptides) (0.500 < %Rank < 2.000) (Table S2). From the *in silico* analysis, 15 unique peptide sequences (8 9-mers, 5 10-mers, and 2 11-mers) were selected according to the following criteria: (i) peptides predicted to bind both H-2K^b^ and HLA-A*0201 and (ii) strong binders with IC_50_ values of <1,000 nM (Table S2). Although H-2K^b^ predominantly prefers octamers, most MHC molecules have a strong preference for 9-mer ligands (35). Thus, eight nonamer-promiscuous H-2K^b^ and HLA-A2 binders were prioritized for the following experimental analysis, none of which have previously been reported.

10.1128/mbio.02666-22.4 TABLE S1 Geographic distributions of HLA-A*0201 and worldwide distributions of C. parvum outbreaks and morbidity/mortality. Download Table S1, DOCX file, 0.09 MB. Copyright © 2023 Wang et al. 2023 Wang et al. https://creativecommons.org/licenses/by/4.0/ This content is distributed under the terms of the Creative Commons Attribution 4.0 International license.

10.1128/mbio.02666-22.5 TABLE S2 Complete list of the peptides selected from C. parvum by *in silico* prediction methods. The binding affinities for H-2K^b^ and HLA-A*0201 were estimated using the NetMHCpan4.0 server. Altogether, 202 8- to 11-mer peptides, including 107 H-2K^b^-restricted peptides and 110 HLA-A*0201-restricted peptides, were predicted for the C. parvum proteins. Fifteen peptides (marked in bold yellow) were predicted for both H-2K^b^- and HLA-A*0201-binding motifs with high estimated binding affinities. The estimated binding affinities for H-2K^b^ and HLA-A*0201 were measured as IC_50_ values in nM units and the peptides were selected with theoretical IC_50_ values ranging from 2.9 to 1,179.35 nM, for which the binding level was strong (rank threshold for strongly binding peptides, %Rank < 0.500) or weak (rank threshold for weakly binding peptides, 0.500 < %Rank < 2.000). Binding affinity values are in nM units and the IC_50_ values are inversely proportional to the affinity. Download Table S2, DOC file, 0.08 MB. Copyright © 2023 Wang et al. 2023 Wang et al. https://creativecommons.org/licenses/by/4.0/ This content is distributed under the terms of the Creative Commons Attribution 4.0 International license.

### The Gp40/15-derived epitopes induced a significant CD8^+^ T-cell response in C57BL/6 mice.

**FIG 1:**
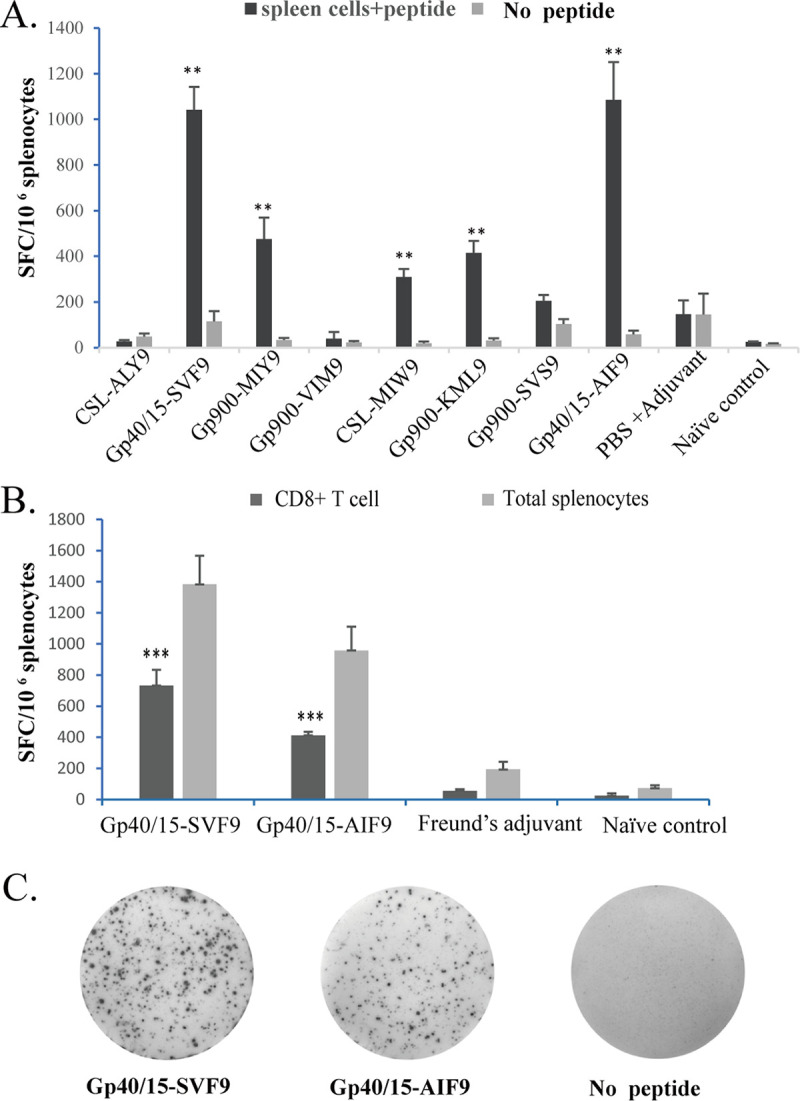
Identification of C. parvum-specific CD8^+^ T-cell epitopes in C57BL/6 mice immunized with oocysts followed by peptide. (A) IFN-γ ELISpot analysis of splenocytes obtained 7 days after C. parvum oocyst and peptide immunization of mice (*n* = 6). Seven days after the last immunization, cells were restimulated with 8 peptides *in vitro*. (B) Splenocytes were isolated with a mouse CD8^+^ T-cell isolation kit (CD8^+^ T cell) or without depletion (Total splenocytes). The peptide-immunized groups were immunized with 50 μg (100 μL) of either peptide packaged with Freund’s adjuvant in a total volume of 200 μL per mouse. The adjuvant controls received 100 μL of PBS and 100 μL of Freund’s adjuvant, and the naive controls were each injected with 200 μL of PBS. (C) Representative ELISpot wells and splenocytes of CD8^+^ T cells isolated from mice immunized with oocysts and peptides. Frequencies of IFN-γ^+^ cells/total CD8^+^ T cells are indicated. Data represent means ± standard deviations (SD), with significance determined using Student’s *t* test (**, *P* < 0.01; ***, *P* < 0.001).

To examine the T-cell responses specific for C. parvum surface antigens, C57BL/6 mice were vaccinated using a strategy of priming with attenuated oocysts and boosting with synthetic peptides (36, 37). Individual peptides were tested, and T-cell-derived IFN-γ was quantified using an ELISpot assay (38) (Fig. 1). Five peptides (Gp40/15-SVF9, Gp900-MIY9, CSL-MIW9, Gp900-KML, and Gp40/15-AIF9) elicited relatively strong responses compared to those in spleen cells without peptides restimulated with spot magnitudes of 1,042, 476, 309, 415, and 1,085 input cells, respectively (*P* < 0.01), whereas the remaining peptides elicited only weak responses (Fig. 1A and Table 1). Two peptides (Gp40/15-SVF9 and Gp40/15-AIF9 [referred to as SVF9 and AIF9 here]) consistently elicited robust IFN-γ responses in a proportion of the total T cells. Given the importance of IFN-γ production in the ELISpot assay, we further investigated SVF9- and AIF9-specific IFN-γ production by CD8^+^ T cells via ELISpot and intracellular cytokine staining assays following three sets of immunizations with the SVF9 or AIF9 peptide vaccines. The results showed that the SVF9 and AIF9 peptides induced significantly higher numbers of IFN-γ spots in both total splenocyte cultures and CD8^+^ T-cell cultures, unlike the splenocytes isolated from the mouse groups immunized with Freund’s adjuvant or the naive negative control (*P < *0.01) (Fig. 1B). Cells stimulated with no peptide or cells from naive mice stimulated with either peptide did not respond to either SVF9 or AIF9. The values of the total IFN-γ-positive (IFN-γ^+^) cells and CD8^+^ T cells are indicated by representative ELISpot wells (Fig. 1C).

**TABLE 1:** Selected peptides used in ELISpot assays and gel filtration and anion-exchange chromatography^*a*^

Epitope (positions)	Peptide name (peptide sequence)	Stability with H-2K^b^	Stability with HLA-A*0201	CD8^+^ T cells with peptide restimulation in ELISpot assays
Gp40/15 (313–321)	Gp40/15-SVF9 (S**V**FAI**F**AA**L**)	++	++	++
Gp900 (16–23)	Gp900-VIM9 (V**I**MNP**L**FS**L**)	++	++	−
CSL (111–119)	CSL-MIW9 (M**I**WHK**S**VN**L**)	++	+	++
Gp900 (815–823)	Gp900-MIY9 (M**I**YDY**N**SG**L**)	++	++	++
Gp900 (807–815)	Gp900-KML (K**M**LDK**Y**TR**M**)	++	−	++
Gp900 (1689–1697)	Gp900-SVS9 (S**V**SGV**F**AT**V**)	++	−	−
Gp40/15 (316–323)	Gp15/40-AIF9 (A**I**FAA**L**FV**L**)	+	−	++
CSL (79–87)	CSL-ALY9 (A**L**YDA**Y**CI**L**)	−	−	−

a For the ELISpot assays, ++ indicates that CD8^+^ T cells from immunized mice elicited a relatively strong response compared to that for spleen cells without restimulated peptides, and − indicates that the peptides elicited only a weak response and no significant difference between spleen cells with and those without restimulated peptides. In the *in vitro* refolding assay, an elution peak representing the pMHC complex in the gel filtration experiments indicated that the peptide was able to bind to the MHC molecule; ++ indicates that the complex was stably eluted under anion-exchange conditions, + indicates that the refolding efficiency was 50 to 60% of that of ++ and that the complex was not stable under anion-exchange conditions, and − indicates that there was no elution peak 2 for the pMHC complex or that it had a much lower peak 2 (less than 80% of peak 1) at 78 to 84 mL in the measurement by Superdex 200 16/600 HiLoad size-exclusion chromatography. The anchor residues of the peptides are shown in bold. All of the peptides came from the C. parvum Iowa II CSL zinc finger protein and the Gp40/15 or Gp900 glycoprotein.

**FIG 2:**
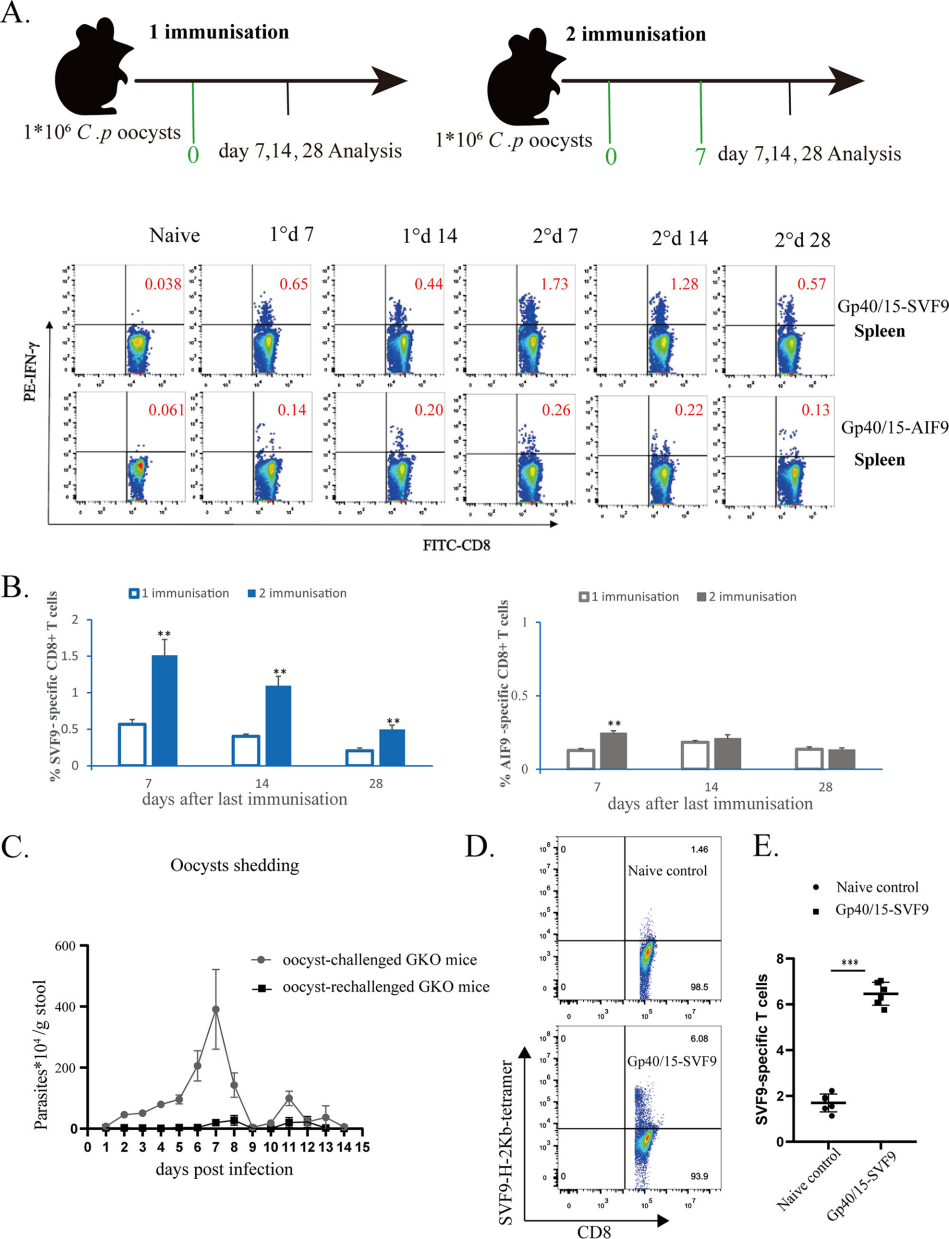
Kinetics of SVF9- and AIF9-specific CD8^+^ T-cell responses following immunization with C. parvum oocysts. (A) C57BL/6 mice were vaccinated either once or twice with C. parvum (*C.p*) oocysts, as shown in the schematic diagram. On days 7, 14, and 28 after the last immunization, SVF9- and AIF9-specific CD8^+^ T-cell responses were quantified in the spleens by peptide stimulation followed by ICS. Representative flow cytometry plots show IFN-γ secretion by CD8^+^ T cells in the spleens of C. parvum oocyst-immunized mice; 1° represents one immunization with C. parvum oocysts, while 2° represents two immunizations with oocysts. FITC, fluorescein isothiocyanate. (B) Data in panel A presented as bar graphs. Empty bars, 1 immunization; filled bars, 2 immunizations (blue for SVF9 and gray for AIF9). **, *P* < 0.01 by a Mann-Whitney test for differences between 1 and 2 immunizations. Experiments were performed at least 3 times, with 4 mice per group. (C) Comparative analysis of oocyst shedding intensities in C. parvum oocyst-challenged and rechallenged GKO mice. (D) Tetramer staining for single-cell isolation of SVF9-specific T cells in representative splenocytes stained with the H-2K^b^–SVF9 tetramer of CD8^+^ T cells isolated from oocyst-infected and naive mice. Frequencies of IFN-γ^+^ cells/total CD8^+^ T cells are indicated. (E) Data from panel D presented as bar graphs, showing differences in peptide-specific CD8^+^ T cells between oocyst-infected and naive mouse immunizations. ***, *P* < 0.001.

Since the Gp40/15-derived peptides SVF9 and AIF9 induced a high level of CD8^+^ T-cell responses after receiving both C. parvum oocysts and peptide vaccines, we wondered whether oocyst infection alone could trigger a similar immunodominant response. Thus, we quantified the responses in the spleen after one or two immunizations with 1 × 10^6^ C. parvum oocysts (Fig. 2A). After a single immunization, SVF9-specific CD8^+^ T-cell responses reached the highest magnitude on day 7 (Fig. 2A and B). The responses were slightly decreased on day 14 as a contraction of the response occurred, but more robust responses were observed after two immunizations with oocysts. Compared with SVF9 peptides, AIF9-specific CD8^+^ T-cell responses were relatively weak and showed no significant differences in primary and secondary responses at 14 and 28 days (Fig. 2A and B). Priming with viable C. parvum oocysts alone was sufficient to provide protective immunity to C57BL/6 mice, and the parasite load was significantly decreased with the second challenge with oocysts administered to IFN-γ knockout (GKO) mice (Fig. 2C). To further characterize these SVF9-specific CD8^+^ T cells, CD8^+^ T cells from the oocyst-immunized mice were stained with the H-2K^b^–SVF9 multimer, and the frequencies of SVF9-specific CD8^+^ T cells were significantly higher than those in naive mice (Fig. 2D and E). Together, these results indicated that immunization with C. parvum oocysts recruits SVF9-specific CD8^+^ T cells to undergo significant peptide-specific T cell responses. These findings confirm that the SVF9 peptide acts as a CTL epitope and is a potential vaccine candidate for C. parvum.

### Vaccination with SVF9 peptides in Freund’s adjuvant elevates protective CTL responses in the small intestine and spleen.

**FIG 3:**
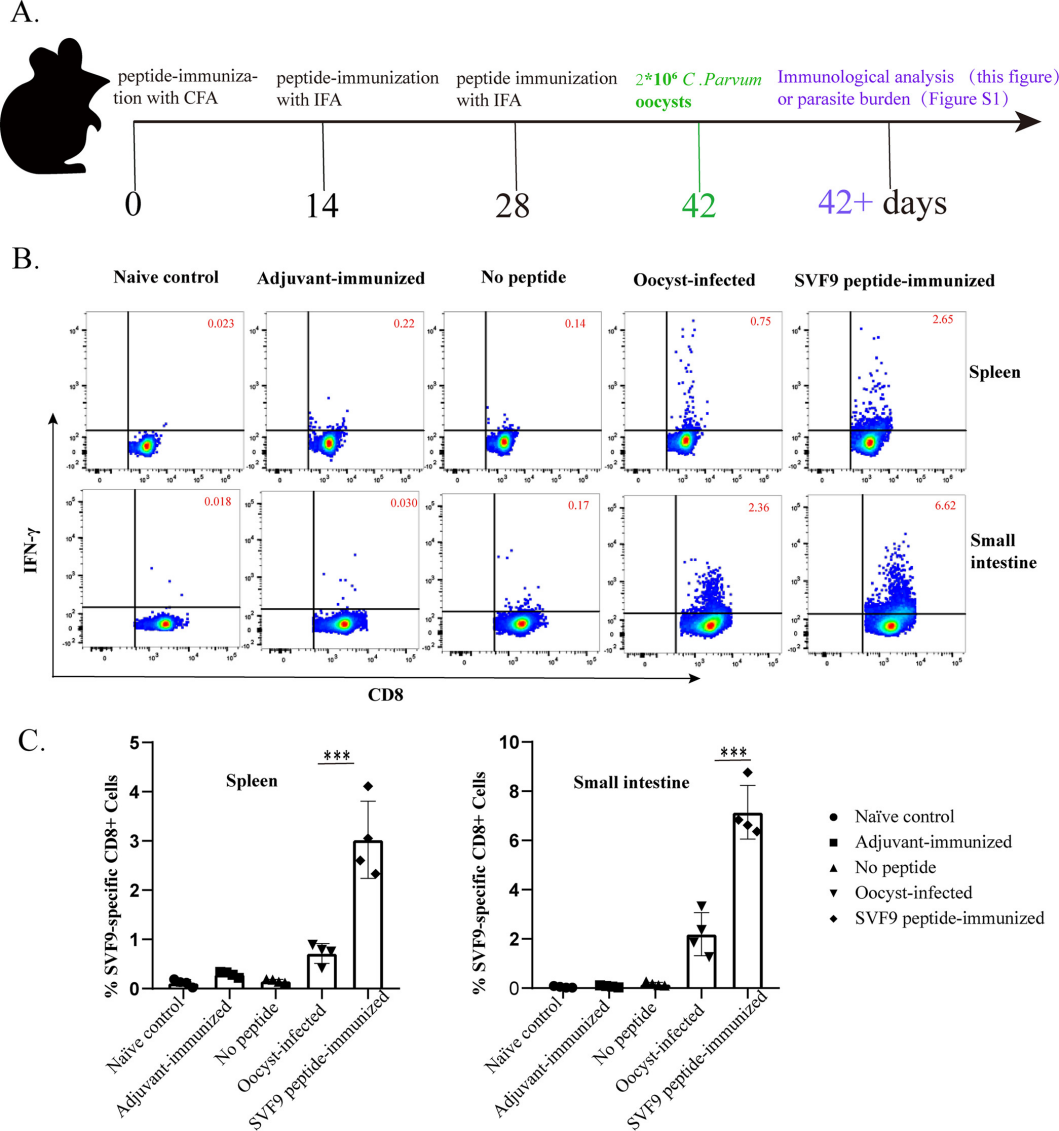
Priming with SVF9 peptide elevates protective CTL responses in the small intestine and spleen. (A) Schematic diagram of the methodology. C57BL/6 mice were primed with the SVF9 peptide and challenged with oocysts. Ten days later, the frequencies and quality of SVF9-specific CD8^+^ T cells in the small intestine and spleen were quantified by ICS. (B) Flow cytometry plots of IFN-γ in spleen cells and IELs. Shown are representative dot plots of CD8^+^ T-cell IFN-γ production following SVF9 peptide restimulation (10 μg/mL) *in vitro*. Values indicate the percentages of CD8^+^ cells within each gate, and data were gated on live CD3^+^ T cells. (C) Analysis of IFN-γ secretion based on the data in panel B. Bars represent the mean values of the percentages of SVF9-specific CD8^+^ cells. Individual data are also shown. Differences between oocyst infection and SVF9-vaccinated immunizations are indicated. Data are means ± SD, and significance was determined using one-way analysis of variance (ANOVA) with Dunnett’s multiple-comparison test (***, *P* < 0.001).

SVF9-specific CD8^+^ T-cell responses are considerably more abundant following *in vitro* restimulation in oocyst-immunized mice. We therefore tested whether vaccination with the SVF9 peptide in Freund’s adjuvant was able to induce a protective CTL response and the benefits of parasite elimination in C57BL/6 mice. As shown in Fig. 3, mice were subcutaneously vaccinated with the SVF9 peptide admixed in complete Freund’s adjuvant (CFA) or incomplete Freund’s adjuvant (IFA) at 2-week intervals before challenge with 2 × 10^6^ oocysts during the immunization protocol (Fig. 3A). Priming with the SVF9 peptide induced higher frequencies of IFN-γ secretion than those of CD8^+^ T cells from both the spleen and small intestine of oocyst-infected mice (*P* < 0.001), and cells from naive or adjuvant-immunized control mice did not respond to SVF9. The antigen-specific response of intraepithelial lymphocytes (IELs) was generally stronger than that of splenocytes, probably due to local site differences in C. parvum (Fig. 3B and C). Using the highly immunogenic SVF9 CTL peptide in Freund’s adjuvant as a model peptide-based vaccine, we investigated whether SVF9 peptide vaccination could prevent C. parvum infection. Next, the number of oocysts per gram (OPGs) was estimated at 1 to 28 days postinfection (dpi). As shown in Fig. S1, all mice inoculated with oocysts became infected. Oocysts were first detected in the feces at 4 dpi by light microscopy and peaked at days 6 to 8 in C57BL/6 mice, with a second level peaking at 12 dpi. Vaccination with the SVF9 CTL peptide could partially attenuate the severity of C. parvum infection in C57BL/6 mice albeit at a low level (Fig. S1). The oocyst shedding intensity in peptide-vaccinated mice was significantly lower at the peak of the oocyst excretion curve (7 dpi) than that in naive control mice (*t *= 10.099; *P* = 0.000). No oocysts were found in the SVF9-immunized group at 15 dpi, suggesting a critical role of T lymphocytes in parasite clearance (11).

10.1128/mbio.02666-22.1 FIG S1 Oocyst shedding of peptide-immunized and naive control mice. Note that oocyst shedding was quantified as oocysts per gram (OPGs) using a hemocytometer from days 1 to 28 postinfection. Download FIG S1, TIF file, 0.3 MB. Copyright © 2023 Wang et al. 2023 Wang et al. https://creativecommons.org/licenses/by/4.0/ This content is distributed under the terms of the Creative Commons Attribution 4.0 International license.

### The conserved SVF9 peptide is stably presented by the H-2K^b^ and HLA-A*0201 molecules.

**FIG 4:**
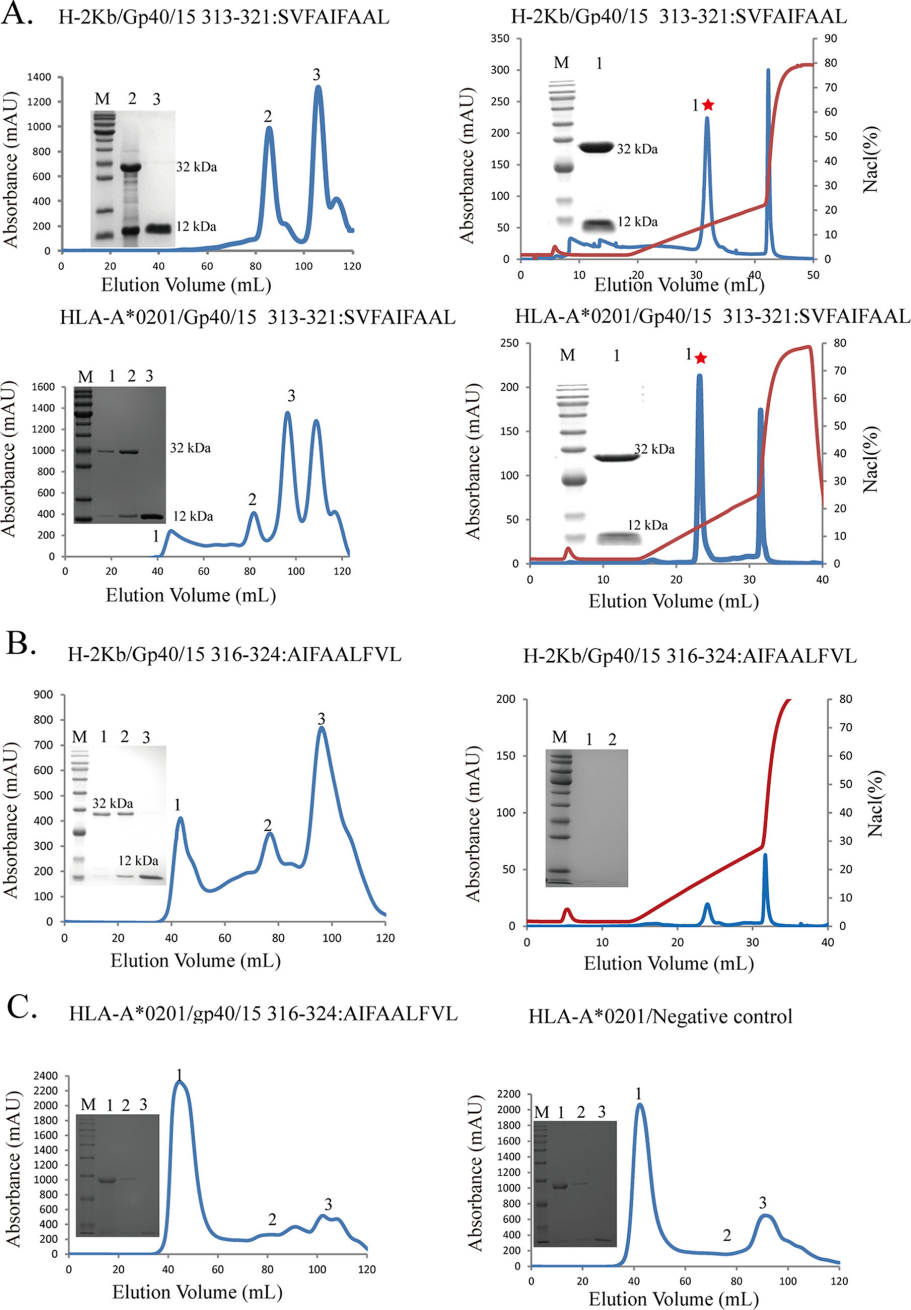
Peptide binding identified by FPLC gel filtration and anion-exchange chromatography with H-2K^b^ and HLA-A*0201. (A) SVF9 peptide formed stable complexes with H-2K^b^ and HLA-A*0201 that survived both purifications. Peak 1 corresponds to the aggregated heavy chain, peak 2 corresponds to the correctly refolded complex, and peak 3 corresponds to excess β2m. (Inset) Reduced SDS-PAGE gel (12%) showing peaks 1, 2, and 3. Lane M contains molecular weight markers (in kilodaltons). Both complexes were eluted at NaCl concentrations of 14.0 to 16.0%. The specific peaks are marked with red asterisks. (B) Peptide AIF9 with H-2K^b^ complexes that tolerated gel filtration but mostly dissociated under anion-exchange chromatography conditions. (C) Nonbinding of the AIF9 peptide with the HLA-A*0201 heavy chain and the negative control from the *in vitro* refolding assays. The sequences of the peptides are shown above their refolding result graphs. mAU, unit of UV absorbance; mS/cm, unit of anion strength.

As the SVF9 peptide is derived from the Gp60 protein, which is relatively less conserved and more likely to be mutated than other *Cryptosporidium* proteins, we assessed the level of homologous peptides from *Cryptosporidium* spp. (Table 2). Interestingly, the SVF9 epitope is highly conserved in C. hominis, C. parvum, C. tyzzeri, and C. felis, which are responsible for over 90% of infections in humans. To evaluate its ability to bind to mouse H-2K^b^ and human HLA-A*0201 heavy chains, the SVF9 peptide was refolded with both H-2K^b^ and HLA-A*0201 using an *in vitro* refolding method and purified by reverse-phase high-performance liquid chromatography (HPLC) (SciLight Biotechnology). The chromatographic results showed that the SVF9 peptide formed stable complexes with both H-2K^b^ and HLA-A*0201 (Fig. 4A), but the AIF9 peptide formed low-stability complexes with H-2K^b^ and could be collected by gel filtration, but the complexes dissociated under Resource-Q anion-exchange chromatography conditions at between 12 and 16 mS/cm (Fig. 4B). The AIF9 peptide could not bind with HLA-A*0201, and no standard chromatographic peaks appeared at 78 to 84 mL by size exclusion chromatography, such as the no-peptide negative control (Fig. 4C).

**TABLE 2:** Gp40/15-SVF9-homologous peptides from *Cryptosporidium* species in humans^*a*^

*Cryptosporidium* species^*b*^	GP40/15-SVF9 homologue	Sequence identities (%)
Tier 1 species		
C. hominis	SVFAIFAAL	100
C. parvum	SVFAIFAAL	100
C. felis	SVFAIFAAL	100
*C. meleagridis*	SVFAI**L**AAL	88
Tier 2 species		
*C. viatorum*	SVFAIFAA**F**	88
*C. canis*	S**LI**A**V**F**VT**F	44
*C. tyzzeri*	SVFAIFAAL	100
Tier 3 species		
*C. fayeri*	SVFAIFAA**F**	88
*C. suis*	S**I**FAIFAA**F**	77

a Note that the GenBank accession numbers are ACQ82740.1 for C. hominis, AAL07532.1 for C. parvum, ABH11920.1 for C. felis, TRY50836.1 for *C. tyzzeri*, ACN87716.1 for *C. fayeri*, AJP62575.1 for *C. viatorum*, AYA71611.1 for *C. suis*, AVV63122.1 for *C. meleagridis*, and QPX49991.1 for *C. canis*.

b Tier 1 species are *Cryptosporidium* spp. that are responsible for over 90% of infections in humans. Tier 2 species are *Cryptosporidium* spp. that have each been reported in at least five human cases. Tier 3 species are *Cryptosporidium* spp. for which confirmed detection has been reported in fewer than five individuals. The mutated residues of the homologous peptides are shown in bold.

**FIG 5:**
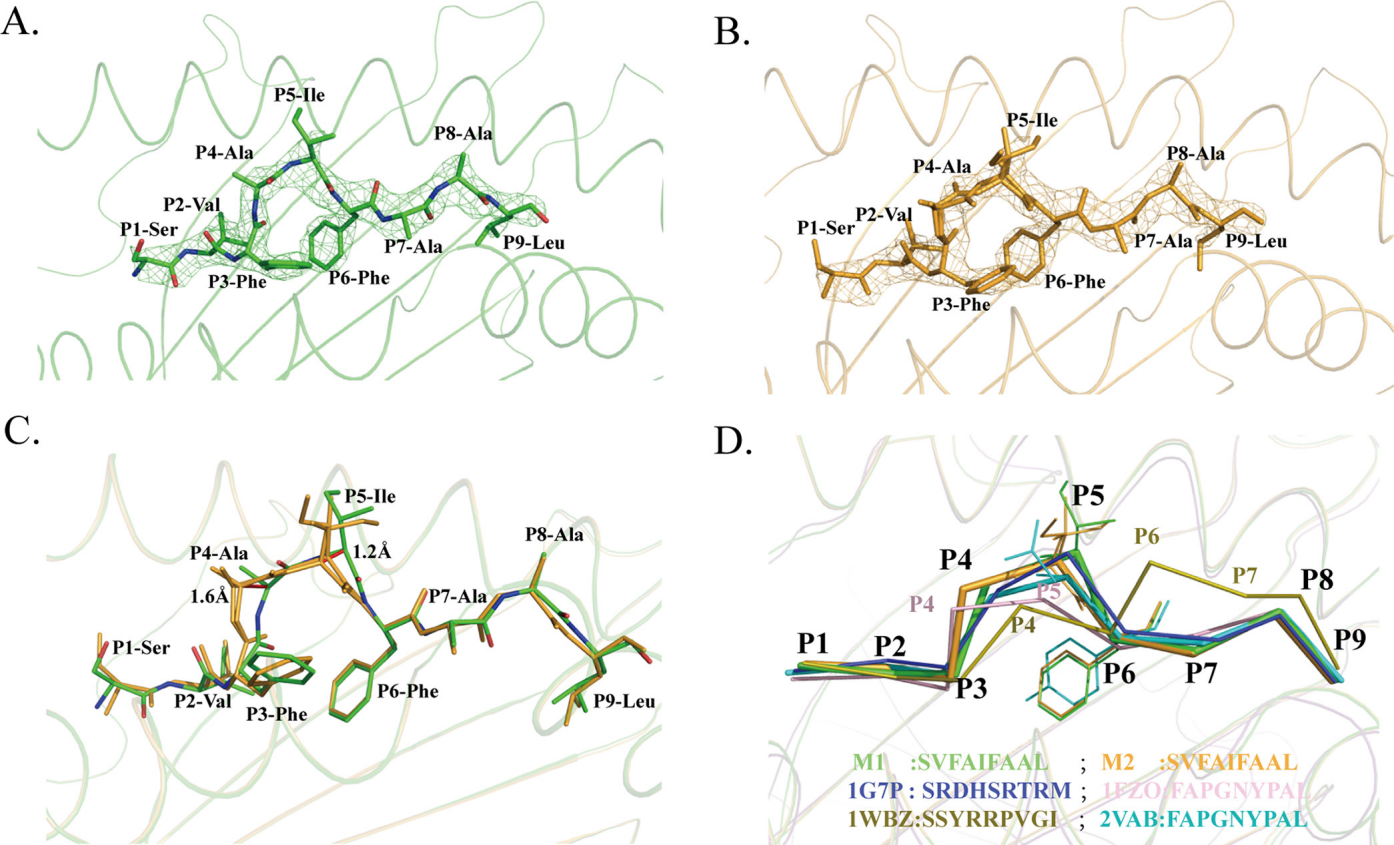
Distinct conformations and electronic densities of SVF9 peptides found in H-2K^b^ M1 and M2. (A and B) Electron density at the 1σ contour level of SVF9 peptides shown in the H-2K^b^ M1 and M2 structures. The electron density maps are clear and indicate that the two distinct peptide conformations are reliable. (C) Detailed comparison of SVF9 peptides shown as a stick model (green, M1; orange, M2). The mismatch of the two peptides is obvious, especially at P4 Ala and P5 Ile. (D) SVF9 peptide conformations (thick sticks) compared to those of other peptides (thin sticks) with distinct conformations presented by H-2K^b^ (blue, PBD accession no. 1G7P; pink, accession no. 1FZO; olive, accession no. 1WBZ; cyan, accession no. 2VAB).

Furthermore, we refolded and crystallized the H-2K^b^–SVF9 complex and solved its structure. H-2K^b^ complexed with SVF9 was crystallized in the P1211 space group with a high resolution of 2.3 Å (Table 3). Within one asymmetric unit, there are two H-2K^b^ molecules, referred to below as M1 and M2. The electron density map was clear for the peptide, indicating a stable and rigid conformation of the SVF9 peptide in the H-2K^b^ cleft (Fig. 5A and B). The comparison showed that the carbon backbones of the SVF9 peptide in M1 and M2 are not coincident, and the distances between the P4 and P5 atoms of the peptides could reach 1.6 Å and 1.2 Å, respectively (Fig. 5C). By comparing all of the other nonapeptides presented by mouse H-2K^b^ molecules (Protein Data Bank [PDB] accession no. 1G7P, 1FZO, 1WBZ, and 2VAB), the M1 peptide conformation was found to be almost identical to that of the molecule under PDB accession no. 2VAB, a dominant CD8^+^ T-cell epitope derived from Sendai virus nucleoprotein (39, 40) (Fig. 5D).

**TABLE 3:** X-ray diffraction data collection and refinement statistics

Parameter	Value(s) for H-2K^b^^*a*^
Data processing statistics	
Space group	P1211
Unit cell parameters	
*a*, *b*, *c* (Å)	60.92, 84.77, 95.24
α, β, γ (°)	90.00, 90, 90.00
Resolution (Å)	50.0–2.3 (2.3–2.34)
No. of reflections	270,761 (28,872)
No. of unique reflections	39,868 (4,091)
Completeness (%)	98.3 (100)
Avg *I*/σ(*I*)	15.259 (1.000)
*R*_merge_ (%)^*b*^	5.9 (63.6)
CC_1/2_ (%)^*c*^	0.999 (0.826)
Refinement statistics	
*R*_work_ (%)^*d*^	22.58
*R*_free_ (%)	25.84
RMSD from ideality^*e*^	
Bond lengths (Å)	0.007
Bond angles (°)	1.468
Avg *B* factor	68.212
Ramachandran plot quality (%)	
Most favored regions	95.60
Allowed regions	4.27
Disallowed regions	0.13

a The numbers in parentheses indicate the highest-resolution shell.

b *R*_merge_ = Σ*_hkl_* Σ*_i_* |*I_i_*(*hkl*) − <*I*(*hkl*)>|/Σ*_hkl_* Σ*_i_ I_i_*(*hkl*), where *I_i_*(*hkl*) is the observed intensity and <*I*(*hkl*)> is the average intensity from multiple measurements.

c CC1/2, percentage of correlation between intensities from random half-datasets.

d *R* = Σ*_hkl_* ‖*F*_obs_| − *k* |*F*_calc_‖Σ*_hkl_* |*F*_obs_|, where *R*_free_ is calculated for a randomly chosen 5% of reflections and *R*_work_ is calculated for the remaining 95% of reflections used for structure refinement.

e RMSD, root mean square deviation.

**FIG 6:**
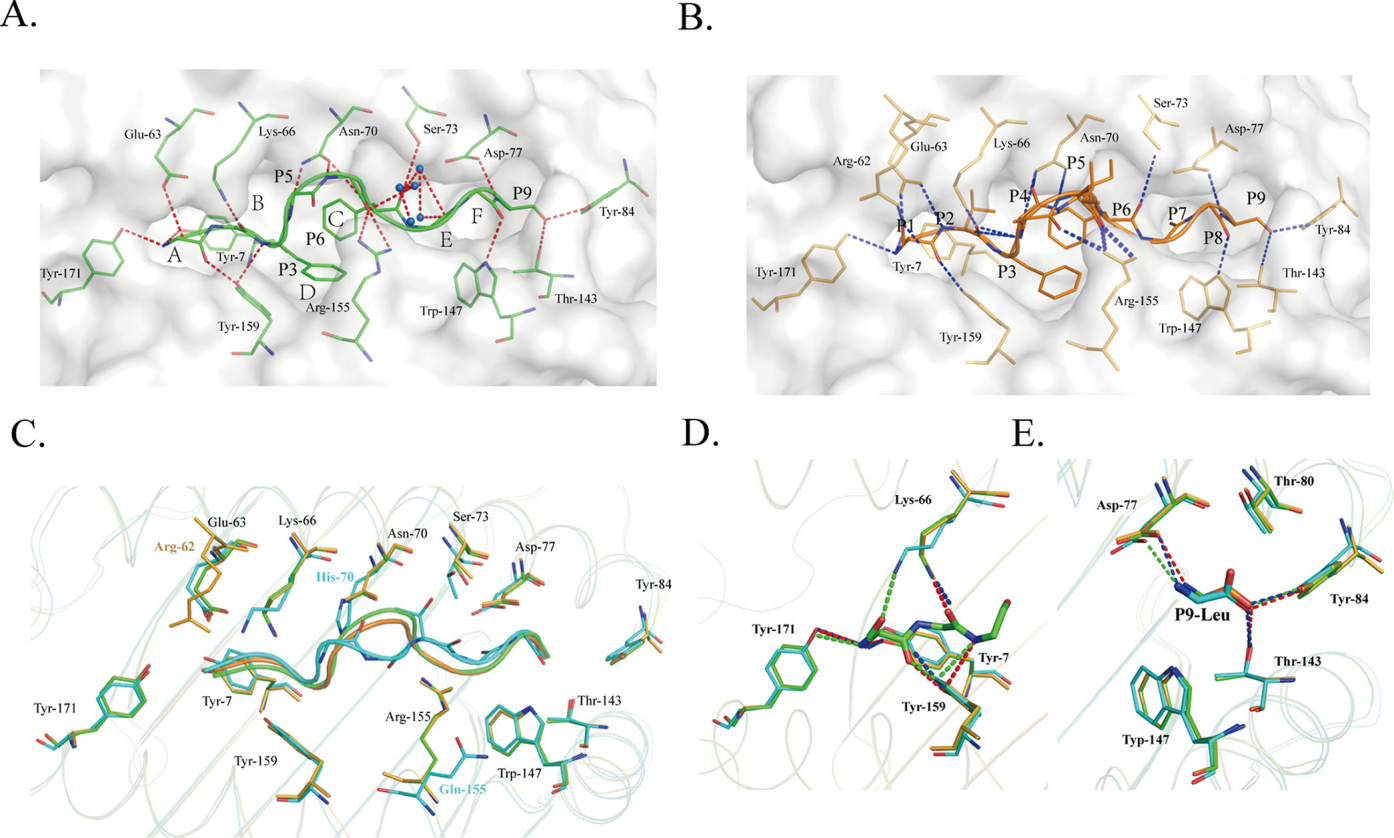
Peptide interactions with H-2K^b^ and HLA-A*0201. (A and B) Hydrogen bonds and water molecules found in H-2K^b^–SVF9 M1 (A) and M2 (B). Surface model P1, P2, P3, P6, and P9 residues in the SVF9 peptide are accommodated by the A, B, D, C, and F pockets, respectively; 5 water molecules in M1 form 5 direct bonds. The blue balls represent water molecules. (C) Comparison of peptide conformations and peptide-MHC surface residues of H-2K^b^ and HLA-A*0201. Detailed MHC surface residues are shown as stick models in M1 (green), M2 (orange), and HLA-A*0201 (cyan). (D) Comparison of the peptide-MHC interactions of P1 Ser, P2 Val, and P3 Phe (the N terminus of PBG) between H-2K^b^ (green and orange) and HLA-A*0201 (PDB accession no. 7RTR) (blue). (E) Comparison of the interactions of P9 Leu in the F pocket (the C terminus of PBG). The hydrogen bonds between the peptides and the pockets are shown as dashed red (M1), blue (M2), and green (HLA-A*0201) lines.

Although the conformations of the SVF9 peptide in M1 and M2 are different, the accommodation of residues by the 6 pockets is roughly the same. The major proportions of P1, P2, P3, P6, and P9 in the SVF9 peptide are accommodated by the A, B, D, C, and F pockets, respectively. The SVF9 peptide bound to the H-2K^b^ cleft via canonical primary anchors of small hydrophobic residues, P2 Val and P9 Leu, characteristics similar to those of H-2K^b^ and HLA-A*0201. The complex structure of H-2K^b^ exhibits a high degree of similarity with the octamer, as a secondary anchor residue (F or Y at position 6) is deeply buried in central pocket C of the H-2K^b^ binding groove. In M1, there are 16 hydrogen bonds between the peptide-binding groove (PBG) and the SVF9 peptide (Fig. 6A), but in M2, there are 19 hydrogen bonds and one salt bridge (Fig. 6B). Moreover, among the water molecules involved in the hydrogen bond network between the PBG and the SVF9 peptide, 5 water molecules form 5 direct hydrogen bonds with the SVF9 peptide in M1, but no water molecules are involved in M2 (Fig. 6A and B). The main differences are found mainly in the middle of the groove. The P4 Ala and P5 Ile residues exhibit double conformations in the M2 molecule. The double main chains of P4 Ala form two hydrogen bonds with Asn70 and Arg155, but no interaction was found for the P4 Ala residue of M1 (Fig. 6B). In addition to hydrogen bonds, the interactions formed by van der Waals forces (VDWs) are also different in the two H-2K^b^ H chains. The total numbers of VDWs are 147 and 160 in M1 and M2, respectively (Table 4). The water molecules of M1 and more hydrogen bonds of M2 could fit into the different PBG conformations to stabilize the distinct peptide conformations. By comparing the peptide-MHC interactions of H-2K^b^ and HLA-A*0201–YLQ–TCR (PDB accession no. 7RTR) (41), the peptide conformations of P1, P2, P3, and P9 are similar to that of HLA-A*0201, and the residues of peptide-MHC interactions are highly conserved, except for Arg155/Gln155 and Asn70/His70 (Fig. 6C). The hydrogen bonds of P1 Ser, P2 Val, P3 Phe, and P9 Leu (the N and C termini of PBG) of the SVF9 peptide are similar to those of a dominant severe acute respiratory syndrome coronavirus 2 (SARS-CoV-2)-derived epitope presented by HLA-A*0201, which can be recognized by a public human TCR (41) (Fig. 6D and E). We believe that the conserved SVF9 peptide can be stably presented by both H-2K^b^ and HLA-A*0201 molecules.

**TABLE 4:** Hydrogen bonds and van der Waals interactions between peptides and peptide-binding domains of M1 and M2 molecules

Complex	Peptide		Hydrogen bond partner		van der Waals contact residues (van der Walls force)^*a*^
	Residue	Atom	Residue	Atom	
Molecule 1	P1 Ser	N	Tyr7	OH	Leu5, Glu63, Lys66, Tyr159, Thr163, Trp167, Tyr171 (24)
		N	Tyr171	OH	
		OG	Glu63	OE2	
		O	Tyr159	OH	
	P2 Val	O	Lys66	NZ	Tyr7, Glu24, Tyr45, Glu63, Lys66, Tyr159 (22)
	P3 Phe	O	Asn70	ND2	Asn70, Arg155, Leu156, Tyr159 (20)
		N	Tyr159	OH	
	P4 Ala				Lys66, Asn70, Arg155 (9)
	P5 Ile	N	Asn70	OD1	Gly69, Asn70, Arg155 (10)
		O	Arg155	NH1	
		O	Arg155	NH2	
	P6 Phe	N	Asn70	OD1	Asn70, Phe74, Gln114, Tyr116, Ser 99, Ser73 (16)
		O	Ser73	OG	
	P7 Ala				Trp147, Glu152, Arg155 (13)
	P8 Ala	O	Trp147	NE1	Asn77, Lys146, Trp147 (10)
	P9 Leu	N	Asp77	OD1	Asp77, Thr143, Trp147, Tyr84, Lys146, Thr80, Lys146 (26)
		O	Tyr84	OH	
		O	Thr143	OG1	
Molecule 2	P1 Ser	N	Tyr7	OH	Tyr7, Arg62, Glu63, Tyr159, Tyr171 (27)
		N	Tyr171	OH	
		OG	Arg62	NH2	
		OG	Glu63	OE2	
		O	Tyr159	OH	
	P2 Val	N	Glu63	OE1	Tyr7, Glu63, Tyr45, Glu24, Lys66, Tyr159 (21)
		O	Lys66	NZ	
	P3 Phe	O	Asn70	ND2	Lys66, Asn70, Tyr159, Leu156, Arg155 (28)
	P4 Ala	O	Arg155	NH1	Lys66, Asn70, Arg155 (10)
		O	Asn70	OD2	
	P5 Ile	O	Arg155	NH1	Asn70, Arg155 (17)
		O	Arg155	NH2	
		N	Asn70	OD1	
	P6 Phe				Asn70, Phe74, Gln114, Tyr116, Ser99, Ser73 (15)
	P7 Ala				Asp77, Arg155, Trp147, Glu152 (12)
	P8 Ala	O	Trp147	NE1	Asp77, Lys146, Trp147 (7)
	P9 Leu	N	Asp77	OD1	Asp77, Thr143, Leu81, Tyr84, Thr80, Lys146, Trp147 (23)
		O	Thr143	OG1	
		O	Tyr84	OH	
		O	Lys146	NZ	
				

a Note that numbers in parentheses are the amounts of van der Waals force. Hydrogen bond (cut-off distance 3.5 Å); Salt bridge(cut-off distance 5 Å); Van der Waals (cut-off distance 4 Å).

### Structure-based epitopes predicted in important *Cryptosporidium* species.

It is noteworthy that some peptides that appear to have the correct peptide-binding motif still bind poorly. For example, peptide CSL-ALY9 (ALYDAYCIL), which has anchor residues that are present in high-affinity peptides (Leu at position 2 and Leu at position 9) (Table 1), binds very poorly to H-2K^b^ and HLA-A*0201 by gel filtration and anion-exchange chromatography (Fig. 4C and Table 1). Furthermore, some peptides have been discovered to bind strongly to class I MHC molecules but cannot yet be recognized by CTLs, such as Gp900-VIM9 (VIMNPLFSL) (Table 1 and Fig. 1). These observations indicate that the rules that predict binding affinity will be more critical.

**FIG 7:**
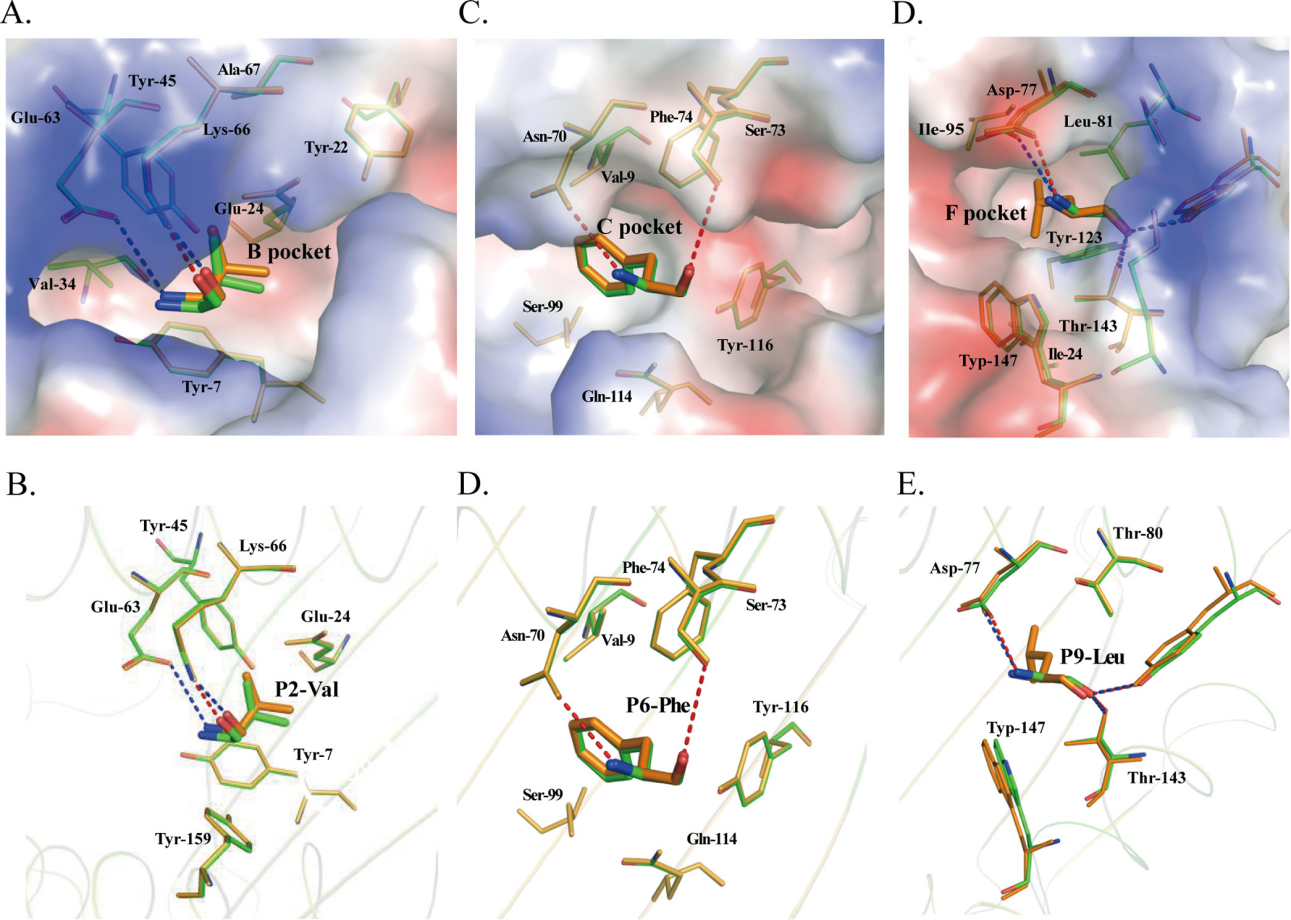
Compositions of the B, D, and F pockets and interactions of H-2K^b^ with SVF9. (A) Electrostatic potential of pocket B with the P2 residue (red, negative; blue, positive; gray, neutral), with the SVF9 peptide in green (M1) and orange (M2); (B) hydrogen bonds and van der Waals interactions between peptides and the B pocket of M1 and M2 molecules; (C) electrostatic potential of pocket C with the P6 residue; (D) interactions between P6 and the C pocket; (E) electrostatic potential of pocket F with the P9 residue; (F) interactions between P9 and the F pocket. The residues comprising the pockets and the residues of bound peptides accommodated by the pockets are shown as stick models. The hydrogen bonds between the peptides and the pockets are shown as dashed red (M1) and blue (M2) lines.

According to the crystal structure of H-2K^b^ with nonapeptides, the B, C, and F pockets accommodated the side chains of P2, P6, and P9, respectively, so they are critical for determining the peptide-binding motif of H-2K^b^. The B pocket is composed of Tyr7, Tyr22, Glu24, Val34, Tyr45, Glu63, Lys66, and Ala67 and shows obvious negatively charged polarity (Fig. 7A). There are two negatively charged residues (Glu24 and Glu63) in the B pocket, and Glu63 forms hydrogen bonds with the main-chain atoms of P2 V in the M2 molecule. The side chain of P2 V stretches to the α1 helix and is inserted into the B pocket, and it is surrounded by uncharged amino acids, except for Glu24 and Glu45 (Fig. 7A and B). Based on our ELISpot and *in vitro* refolding assays, P2 I/V/M is suitable for binding the B pocket, and P2 R/A/G/S could also form stable complexes that survived the strong ionic buffer during anion exchange (42, 43). The C pocket is composed of Val9, Asn70, Ser73, Phe74, Ser99, Gln114, and Tyr116. The main chain of P6 Phe forms two hydrogens with Asn70 and Ser73 of the α1 domain and shows hydrophilic and neutral properties (Fig. 7C and D). P6 F/Y/R/P/L/N/S is suitable for binding the C pocket based on our results and resolved structures, as is P6 I/V/K/H, with similar properties (42, 43). The F pocket is composed of the conserved residues Tyr84, Tyr123, Thr143, Lys146, and Trp147 as well as the less conserved residues Asp77, Thr80, Leu81, Ile95, and Ile124 (Fig. 7E and F). Our results and those from previous studies confirmed that P9 L/M/V/I/A is the most suitable anchor residue for the F pocket of H-2K^b^. In addition, the upward-stretching side chains of P5(I) interact with the TCRs and form many VDWs with the positively charged amino acid Arg155, so P5 of the nonapeptide strongly prefers an amino acid with a large benzene ring or a long side chain. Thus, on the grounds of the results of our structural and functional studies, the 9-mer peptide-binding motif of H-2K^b^ was preliminarily determined to be X-(I/V/M/L/A/G/R/S)-X-X-(Y/F/W/L/I/K/N/T)-(F/Y/R/P/N/S/K/H/L/I/V)-X-X-(L/M/V/I/A).

**FIG 8:**
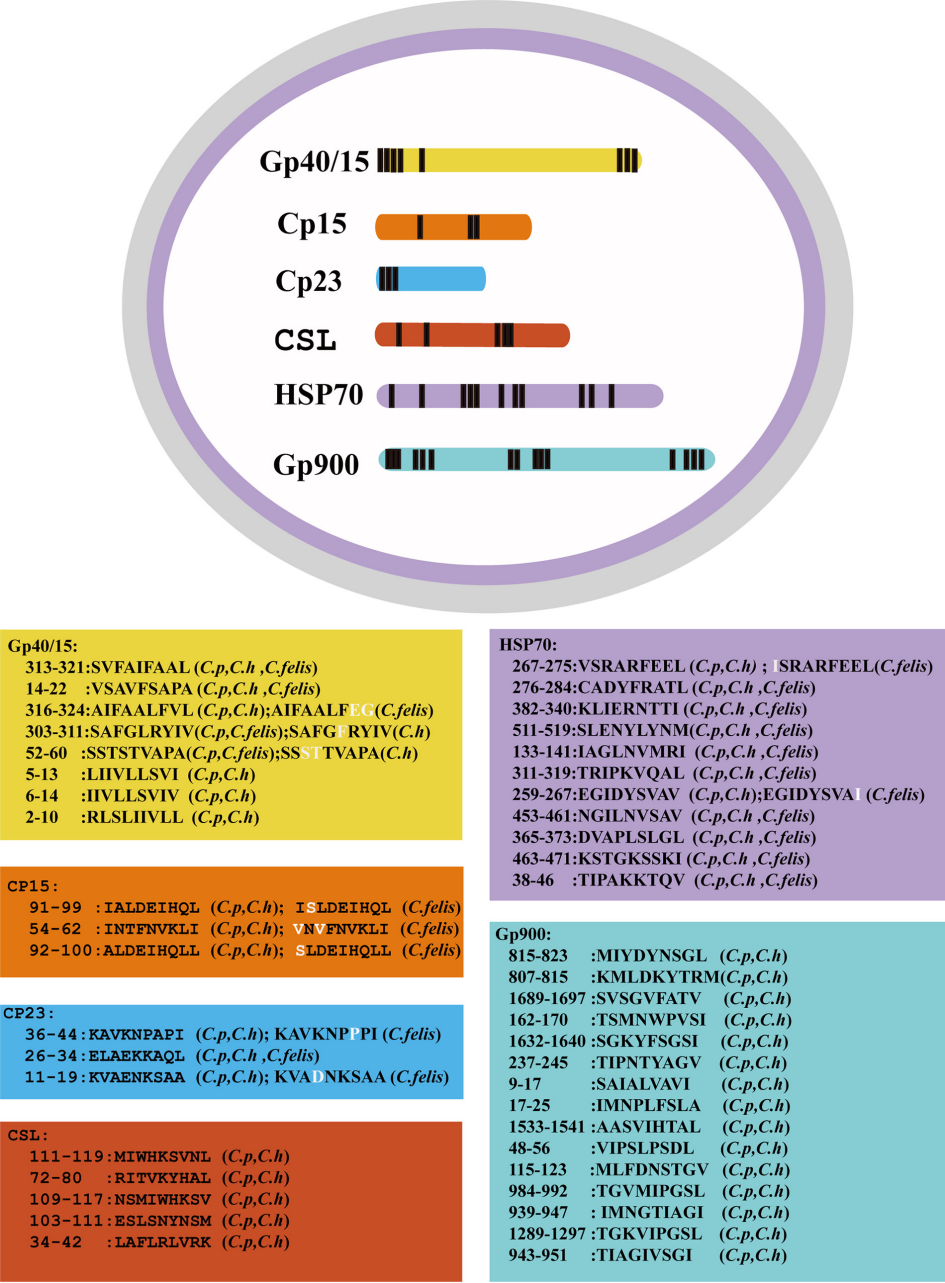
Distribution of H-2K^b^-restricted nonapeptides in important *Cryptosporidium* species. Shown are the results of genome-wide scanning for H-2K^b^-restricted nonapeptides in C. parvum, C. hominis (*C.h*), and C. felis. GenBank accession numbers are as follows: ACQ82740.1 (C. hominis), AAL07532.1 (C. parvum), and ABH11920.1 (C. felis) for Gp40/15; XP_666956.1 (C. hominis), AAA28294.1 (C. parvum), and KAF7458128.1 (C. felis) for Cp15; AEJ22864.1 (C. hominis), AAN31184.1 (C. parvum), and KAF7456923.1 (C. felis) for Cp23; AIT12349.1 (C. hominis), AGS42174.1 (C. parvum), and AKM20838.1 (C. felis) for HSP70; CUV04935.1 (C. hominis) and EAK89290.1 (C. parvum) for CSL; and OLQ18067.1 (C. hominis) and AAC98153 (C. parvum) for Gp900. Forty-five nonapeptides derived from the Gp40/15, Cp23, Cp15, HSP70, CSL, and Gp900 proteins were selected based on the modified motif X-(I/V/M/L/A/G/R/S)-X-X-(Y/F/W/L/I/K/N/T)-(F/Y/R/P/N/S/K/H/L/I/V)-X-X-(L/M/V/I/A).

Based on the preliminary motif described above, we screened the main surface and apical complex protein sequences of C. parvum, C. hominis, and C. felis. There were 49 9-mer epitope peptides that were predicted to fit the motif, including Gp40/15, Cp15, Cp23, CSL, Gp900, and 70-kDa heat shock protein (HSP70). Forty-two of them were completely conserved in C. parvum and C. hominis, and 12 were completely conserved in all three species (Fig. 8; Table S3). HSP70, Cp23, Cp15, and Gp40/15 origin peptides are more conserved than those of CSL and Gp900. HSP70 is highly conserved not only in C. hominis and C. parvum but also in many other *Cryptosporidium* species such as C. meleagridis, C. felis, C. canis, C. ubiquitum, C. cuniculus, C. viatorum, C. bovis, and C. xiaoi. None of the other peptides that we predicted here have been deposited in the Immune Epitope Database (IEDB).

10.1128/mbio.02666-22.6 TABLE S3 Complete list of nonapeptides selected from C. parvum, C. hominis, and C. felis based on the preliminary motif. Note that the GenBank accession numbers are as follows: ACQ82740.1 (C. hominis), AAL07532.1 (C. parvum), and ABH11920.1 (C. felis) for Gp40/15; XP_666956.1 (C. hominis), AAA28294.1 (C. parvum), and KAF7458128.1 (C. felis) for Cp15; AEJ22864.1 (C. hominis), AAN31184.1 (C. parvum), and KAF7456923.1 (C. felis) for Cp23; AIT12349.1 (C. hominis), AGS42174.1 (C. parvum), and AKM20838.1 (C. felis) for HSP70; CUV04935.1 (C. hominis) and EAK89290.1 (C. parvum) for CSL; and OLQ18067.1 (C. hominis) and AAC98153 (C. parvum) for Gp900. Download Table S3, DOC file, 0.06 MB. Copyright © 2023 Wang et al. 2023 Wang et al. https://creativecommons.org/licenses/by/4.0/ This content is distributed under the terms of the Creative Commons Attribution 4.0 International license.

## DISCUSSION

### Vaccination with C. parvum oocysts and Gp40/15-SVF9 synthetic peptides elicits peptide-specific CTL responses.

Adaptive immune responses have been implicated as being important mechanisms of parasite-induced protection, and CD4^+^ Th1 cells and CD8^+^ cytotoxic T lymphocytes (CTLs) are two major subsets of cells mediating protective immunity (10, 17, 44–51). As described in detail in Results, splenocytes isolated from C. parvum-infected and peptide-immunized mice secreted IFN-γ following *ex vivo* restimulation with soluble peptides (Fig. 1A). Two peptides (SVF9 and AIF9) induced significantly higher numbers of IFN-γ spots in both total splenocyte cultures and CD8^+^ T-cell cultures (Fig. 1B). The peptides induced significantly higher levels of IFN-γ production in total splenocytes than in CD8^+^ T-cell cultures, natural killer (NK) cells, dendritic (DC) cells, or CD4^+^ T cells, as well as CD8^+^T cells as the most likely explanation (44, 52, 53). Vaccination with C. parvum CTL peptides in adjuvant elevated SVF9-specific CD8^+^ T-cell responses but did not ensure long-term effectiveness in providing adequate protective immunity (Fig. 3; see also Fig. S1 in the supplemental material). Using the natural Gp40/15 protein-flanking residues to extend the minimal CTL peptide to a 30-amino-acid (aa)-long peptide or supplementation with a CD4^+^ Th peptide may rescue the function of SVF9-induced CD8^+^ T cells and enhance the CTL response to C. parvum (54). Far from perfect, by utilizing IFN-γ as a readout of our assays, we cannot formally exclude other cytokines or cellular markers that may serve as additional signatures of protection.

The SVF9 and AIF9 peptides overlapped and were derived from amino acids 313 to 321 and 316 to 324 of the Gp40/15 protein, respectively. Although similar in terms of their peptide sequences, with Val or Ile at position 2 and Leu at position 9, the AIF9-specific CD8^+^ T-cell response was relatively weak due to the different conformations at position 5 (P5). According to the crystal structure of H-2K^b^ with the SVF9 peptide, P5 of the nonapeptide strongly preferred an amino acid with a large benzene ring or a long side chain, whereas the AIF9 peptide has a P5(A) without a side chain. To determine the primary role of the P5 residue, P5(I) of SVF9 was replaced by alanine (A), and the results confirmed our hypothesis. The substitution of the P5 residue cannot affect peptide binding as the P5 Ala mutant peptide could efficiently form a complex with H-2K^b^. The standard chromatographic peak appeared in the fractions at elution volumes of 78 to 84 mL (marked with red asterisks), similar to the wild-type SVF9 peptide (Fig. S2A). However, restimulation with the P5 Ala mutant peptide resulted in relatively weak IFN-γ production by CD8^+^ T cells in an intracellular cytokine staining assay (Fig. S2B).

10.1128/mbio.02666-22.2 FIG S2 Comparison of SVF9 and the P5 Ala mutant peptide by gel filtration chromatography and ICS. (A) Affinity of binding of the SVF9-P5(A) peptide to H-2K^b^ by *in vitro* refolding assays and gel filtration chromatography. The P5(A) peptide formed a stable complex with H-2K^b^, similar to the wild-type SVF9 peptide, as the standard chromatographic peak appeared in the fractions at elution volumes of 78 to 84 mL (marked with red asterisks). (B) Flow cytometry plots of IFN-γ in spleen cells. Cells of SVF9 peptide-vaccinated mice were isolated and restimulated *in vitro* with SVF9 and P5(A) mutant peptides (10 mg/mL). Figures are representative of data from 1 of 3 experiments with 3 mice/group/experiment. Download FIG S2, TIF file, 1.2 MB. Copyright © 2023 Wang et al. 2023 Wang et al. https://creativecommons.org/licenses/by/4.0/ This content is distributed under the terms of the Creative Commons Attribution 4.0 International license.

### Structural analyses of human public TCRs recognizing the dominant SVF9 epitope presented by H-2K^b^.

Based on our analysis and previous studies, the peptide-binding motif of H-2K^b^ partly overlaps HLA-A*0201, one of the most prevalent HLAs in the global population, which also prefers L or M at position 2 and I or V at the C terminus (33). According to data from the Allele Frequency Net Database (http://www.allelefrequencies.net) (34), the HLA-A*0201 allele is widely distributed in South America, North America, Europe, Africa, and many other countries and regions. For example, in Bolivia and Peru in South America, the gene frequency is 0.6667, and 85.7% of individuals have the allele. Although the distributions of C. parvum subtype families in humans are distinct in different areas and under different socioeconomic conditions (1), C. parvum and C. hominis are responsible for over 90% of infections in humans, and they are responsible for almost all cryptosporidiosis outbreaks investigated (55).

**FIG 9:**
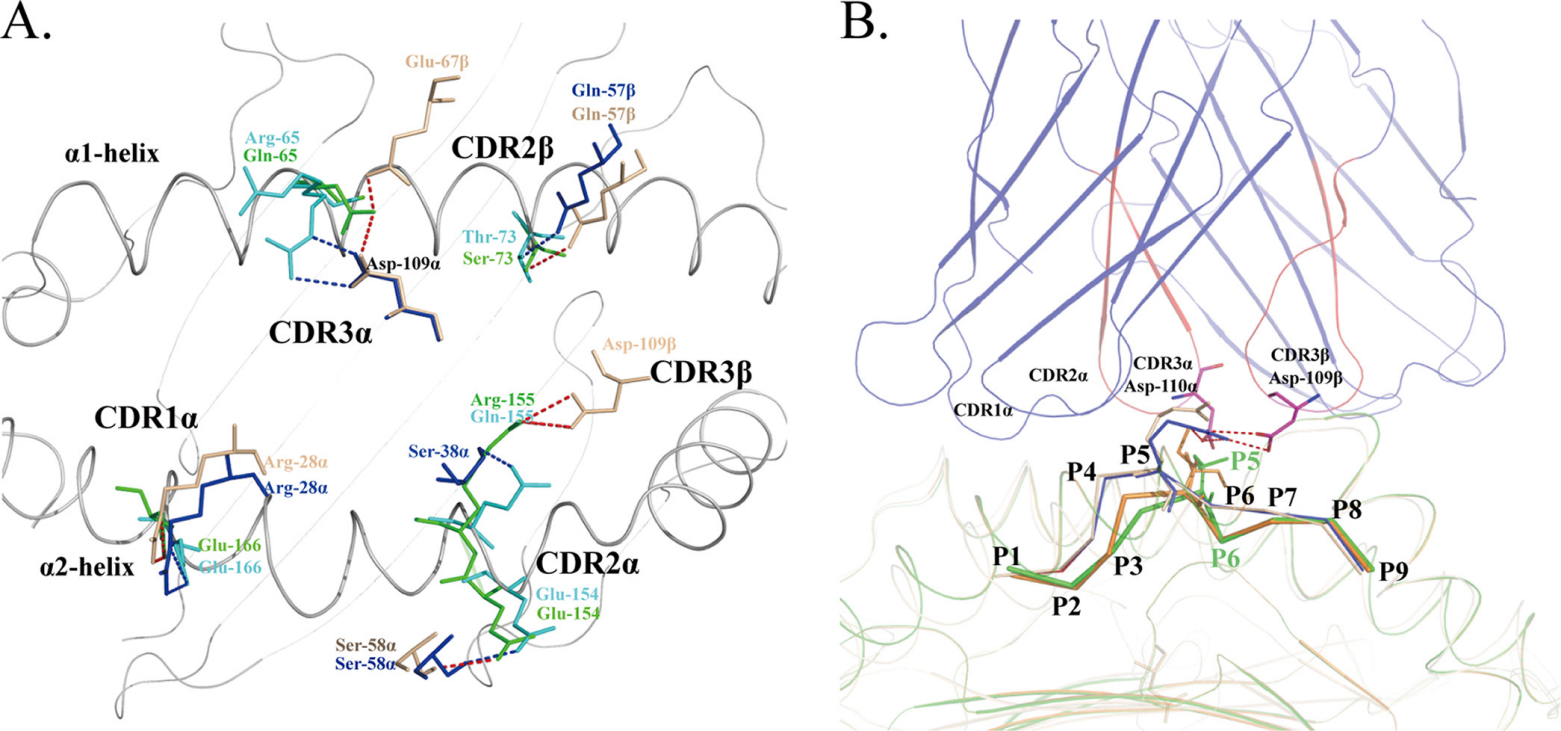
Analyses of contact between human HLA-A*0201 and the public TCR and modeling of the H-2K^b^–TCR complex. (A) Contacts between the public TCR and MHC surface residues on the tops of the helices. Models of the structures of the H-2K^b^–TCR complex (pink sticks), the HLA-A*0201–TCR complex (blue sticks), H-2K^b^ surface residues (green sticks), HLA-A*0201 (cyan sticks), and H-2K^b^ molecule α1 and α2 domains (white cartoon) are shown. The hydrogen bonds and interaction residues are highly conserved, except for Arg155/Gln155 and Arg65/Gln65. (B) Superposition of the HLA-A*0201–YLQ and H-2K^b^–SVF9 peptide structures. In comparison with HLA-A*0201 without the TCR (PDB accession no. 7RTD) (pink), the P5(I) residues of the SVF9 peptides (green in M1 and orange in M2) have conformations more similar to those of P5(R) presented by the HLA-A*0201–TCR complex (PDB accession no. 7RTR) (blue). Asp109β and Asp110α in the conserved motif form two hydrogen bonds with the YLQ peptide, also showing the interaction of the CDR3β and CDR3α loops (red) with the peptide. Interactions are also shown in Table S4.

Can the dominant SVF9 epitope be recognized by human T cells? The molecular recognition of the TCR and pMHC is at the heart of immunology. Although the TCR is specific for the pMHC, it is also “cross-reactive” with many MHC molecules (56). Protein-protein interactions are governed mainly by van der Waals interactions, hydrogen bonds, and salt bridges, similar to TCRs and MHC molecules (56, 57). With a model of the structure of the H-2K^b^ human public TCR presenting the SVF9 peptide, based on modeling from the HLA-A*0201–public TCR complex (PDB accession no. 7RTR), we found that the Vα domain of the TCR lies mainly over the α2 helix of MHC I, and the Vβ domain lies mainly over the respective α1 MHC helices (Fig. 9A). Of note, the contacts of complementarity determining region (CDR), such as CDR1α, CDR2α, and CDR2β among the TCR-pMHC (H-2K^b^ and HLA-A*0201) complexes were similar (Table S4). The conserved contacts between the TCR V regions and MHC helices are flat landscapes that are a secondary consequence of CDR3-peptide interactions (56). The most variable regions of the TCR (CDR3) are positioned in the center of the binding interface where they contact the peptides, and the P5 residues of the peptides are surrounded by the CDR3α and CDR3β loops. The spatial conformation of P5(I) was found to be almost identical to that of P5(R) of HLA-A*0201–TCR (PDB accession no. 7RTR), and Asp109β and Asp110α in the conserved motif of the public TCR seem to exhibit a preference for recognizing the dominant SVF9 epitope (41) (Fig. 9B). This hypothesis has also been supported by functional studies with ELISpot assays and ICS (Fig. 1 and 3). Obviously, the conformations of the prominently exposed P5(R) in the structure under PDB accession no. 7RTR, P5(I) in SVF9, P5(K) in CSL-MIW9, P5(Y) in Gp900-MIY9, and P5(K) in Gp900-KML all have relatively long side chains (Table 1), which can interact with polymorphic TCRs. However, the centers of the negative peptides are likely to be similar to those (PDB accession no. 1QEW, 1QR1, and 3MR9) presented by HLA-A*0201. Similarly, P5(P) in Gp900-VIM9, P5(V) in Gp900-SVS9, and P5(A) in CSL-ALY9 may have the same location and do not assume specific conformations to make stabilizing contacts with the TCRs (58) (Fig. S3).

10.1128/mbio.02666-22.3 FIG S3 Peptide conformations found in P5(P/V/A) presented by HLA-A*0201. Note the detailed comparison of P5(P/V/A) shown as a stick model [wheat, P5A; blue, P5P; yellow, P5(V) (PDB accession no. 3MR9, 1QEW, and 1QR1, respectively)]. Download FIG S3, TIF file, 2.8 MB. Copyright © 2023 Wang et al. 2023 Wang et al. https://creativecommons.org/licenses/by/4.0/ This content is distributed under the terms of the Creative Commons Attribution 4.0 International license.

10.1128/mbio.02666-22.7 TABLE S4 Contacts between the public TCR and HLA-A*0201–YLQ and the H-2K^b^ complex. Abbreviations: HB, hydrogen bond (cutoff distance of 3.5 Å); SB, salt bridge (cutoff distance of 5 Å); VDW, van der Waals (cutoff distance of 4 Å). Download Table S4, DOCX file, 0.02 MB. Copyright © 2023 Wang et al. 2023 Wang et al. https://creativecommons.org/licenses/by/4.0/ This content is distributed under the terms of the Creative Commons Attribution 4.0 International license.

Another interesting phenomenon is that the peptide in the M2 molecule of H-2K^b^–SVF9 exhibits double conformations (Fig. 5). Does the flexibility of P4 and P5 in the center of the SVF9 peptide increase immunogenicity? Perhaps in the context of a peptide that binds well, increased flexibility is more immunogenic, but in the context of a poorly binding peptide, increased flexibility does not increase immunogenicity (58). Notably, SVF9 could be an immunogenic epitope based on its ability to induce peptide-specific CD8^+^ T-cell responses upon *in vivo* functional validation (Fig. 1 to 3). The amino acid sequences of the SVF9 epitope are highly conserved in C. hominis, C. parvum, C. felis, and *C. tyzzeri* (Table 2), which will be useful for designing cross-protective vaccines against these species.

In brief, we provide functional and structural analyses of the dominant C. parvum epitope presented by H-2K^b^ and HLA-A*0201. Further work is needed to improve the immunogenicity of Gp40/15-based vaccines as well as other *Cryptosporidium* proteins involved in the infection and invasion of host cells, such as the rhoptry proteins and the MEDLE family of secretory proteins (59–61). Developing cross-protective vaccines for use in livestock and reducing oocyst shedding would offer new options to combat cryptosporidiosis and have a significant impact on public health.

## MATERIALS AND METHODS

### Animals and materials.

C57BL/6 female mice were purchased from Beijing Vital River Laboratory Animal Technology Co., Ltd., People’s Republic of China. The animal handling and experimental procedures were carried out in compliance with the recommendations of the guide for the care and use of laboratory animals of the Ministry of Health, People’s Republic of China (62). The experimental protocol was approved by the Institutional Animal Care and Use Committee of Zhoukou Normal University on 10 July 2018 (authorization no. IACUC-henau-20180710). Molecular biology kits and enzymes were purchased from TaKaRa Biotechnology, Dalian, People’s Republic of China. Oxidized glutathione, reduced glutathione, l-arginine hydrochloride, and guanidine hydrochloride were purchased from Amresco BioLabs (Solon, OH). Mouse IFN-γ ELISpot Plus (HRP [horseradish peroxidase]) was purchased from MabTech (Cincinnati, OH), and the MojoSort mouse CD8^+^ T-cell isolation kit was purchased from BioLegend (San Diego, CA).

### Prediction, synthesis, and treatment of epitope peptides.

To identify the potential H-2K^b^ allele-specific peptides from C. parvum, a computer-based program was used via an updated version of the NetMHCpan4.0 server (https://services.healthtech.dtu.dk/service.php?NetMHC-4.0) (32). The peptides were synthesized and purified to 90% purity by reverse-phase high-performance liquid chromatography (HPLC) with mass spectrometric analysis (SciLight Biotechnology, Beijing, People’s Republic of China). After synthesis, the peptides were stored in lyophilized aliquots at −80°C and dissolved later in dimethyl sulfoxide. Peptides were diluted in phosphate-buffered saline (PBS) and tested individually at 10 μg/mL for the ELISpot assays and T-cell stimulations.

### Macromolecule production and *in vitro* refolding assays.

The recombinant proteins were expressed as inclusion bodies and purified as described previously (63). The pMHC I complexes (H-2K^b^– mouse β2m (mβ2m)–peptide and HLA-A*0201–human β2m (hβ2m)–peptide) were prepared by refolding assays as described previously, with some modifications (64). Briefly, the MHC I heavy chain and β2m inclusion bodies were separately dissolved in a solution containing 50 mM Tris-HCl (pH 8.0) and 6 M Guanidine-HCl. The MHC I heavy chain (H-2K^b^ or HLA-A*0201), mβ2m or hβ2m, and the peptide, at a 1:1:3 molar ratio, were refolded in refolding buffer (100 mM Tris-HCl [pH 8.0], 2 mM EDTA, 400 mM l-arginine HCl, 0.5 mM oxidized glutathione, and 5 mM reduced glutathione) at 277 K by gradual dilution. After 8 to 12 h of incubation at 4°C, the soluble portions of the complexes were concentrated and then purified by size exclusion chromatography on a Superdex 200 16/600 HiLoad column and by Resource-Q anion-exchange chromatography (GE Healthcare, Stockholm, Sweden).

### Immunizations.

Female C57BL/6 mice (6 to 8 weeks old; 6 per group) were housed under specific-pathogen-free conditions at 25°C with autoclaved food and water provided *ad libitum*. Next, the mice were divided into the following groups: the peptide-immunized groups, the oocyst infection group, the Freund’s adjuvant control group, and the naive control group. C. parvum oocysts collected from infected calves were propagated in C57BL/6 interferon gamma knockout (GKO) mice (stock no. 002287; Jackson Laboratories, USA), which were kindly provided by Yurong Yang (Henan Agricultural University, Zhengzhou, People’s Republic of China). C57BL/6 mice were subcutaneously immunized three times with the mixed peptide at 10- to 14-day intervals. The mixed-peptide vaccine administered to each mouse consisted of 50 μg of polypeptides dissolved in PBS and emulsified in complete Freund’s adjuvant (CFA) for the primary immunization or incomplete Freund’s adjuvant (IFA) for the booster immunization in a total volume of 200 μL. The Freund’s adjuvant-immunized group was given 100 μL Freud’s adjuvant and 100 μL PBS per mouse. Each vaccine solution was emulsified before administration. Two weeks after the last immunization, mice were challenged with 2 × 10^6^ oocysts for the quantitation of fecal parasite loads, four mice from each group were sacrificed, and their splenocytes and small intestine intraepithelial lymphocytes (IELs) were isolated for ELISpot assays and intracellular cytokine staining.

### ELISpot assay.

The identification of the peptide-specific CTL epitopes was performed using the IFN-γ ELISpot assay (product code 3321-4HPT-2; Mabtech, BD Biosciences) according to the manufacturer’s instructions. Single-cell suspensions were made from the spleens of the peptide-immunized, adjuvant-immunized, and naive mice. Erythrocytes were lysed by ammonium chloride treatment. The lymphocytes were seeded in ELISpot plates at 1 × 10^6^ cells/well and stimulated with each peptide at a final concentration of 10 μg/mL for 12-48 h at 37°C. Cells with nonspecific concanavalin A (ConA) (10 μg/mL) (Sigma) stimulation served as a positive control, and naive mice served as a negative control. All antibodies and reagents used for the ELISpot assay were obtained from Mabtech. An automated ELISpot reader (AID, Germany) was used to count the spots using RAWspot technology for multiplexing at the single-cell level. Spot-forming cells (SFCs) were adjusted by subtracting the average negative values and expressed as SFCs per 10^6^ splenocytes. A positive response was defined as having at least 100 SFCs/10^6^ input cells. Cells were plated in at least 3 replicate wells under each condition. The results were quantified as SFCs per 10^6^ murine splenocytes (65).

### Intracellular cytokine staining.

Single-cell suspensions of murine IEL cells were isolated as previously described (66). Briefly, the intestines were removed, and the feces in the intestines were cleared by holding the intestines with forceps and flushing them with a syringe filled with calcium- and magnesium-free 1× Hanks’ buffered salt solution (HBSS) (Gibco). The pieces of the intestine were incubated in 15 mL of digestion solution (HBSS containing 5 mM EDTA and 1 mM dithiothreitol [DTT]) at 37°C for 20 min under slow rotation. Next, the cells were further purified on a discontinuous Percoll gradient. A total of 2 × 10^6^ IELs or splenocytes were prepared in 24-well plates and incubated with a final concentration of 10 μg/mL of peptides and 21 μg/mL of anti-CD28 (clone 37.51) in the presence of brefeldin A (eBioscience) for 5 to 6 h at 37°C with 5% CO_2_. Following stimulation, cells were first stained with BD fixable viability dye 510 and surface stained for 30 min with CD4 (clone GK1.5), CD3 (clone 17A2), and CD8 (clone 53-6.7) (BioLegend) antibodies. Next, the cells were fixed and permeabilized using BD Cytofix/Cytoperm solution (BD Biosciences) and intracellularly stained with anti-IFN-γ–phycoerythrin (PE) (clone XMG1.2) or mouse isotype IgG1-PE (clone MOPC-21) (all from BioLegend) for an additional 30 min. Samples were washed, resuspended in PBS, and acquired on a BD Fortessa instrument (BD Biosciences). Data were analyzed using FlowJo 10.8 (TreeStar, Inc.) and graphically represented using GraphPad Prism V8 (GraphPad Software).

### Quantitation of fecal parasite loads.

C57BL/6 mice were infected by gavage with 2 × 10^6^ C. parvum oocysts followed by peptide immunizations with CFA or IFA. Fecal samples were collected at 1 to 28 days postinfection. The samples were analyzed for parasite loads as oocysts per gram (OPGs) using a hemocytometer as previously described (67, 68). Briefly, *Cryptosporidium* oocysts were isolated from fecal samples using Sheather’s sugar flotation method, and sampling for each group was repeated three times to calculate an average value. The number of OPGs was estimated according to the number of oocysts counted and a curve diagram of oocyst excretion.

### Crystallization and data collection.

The SVF9 peptide of C. parvum (GenBank accession no. AAL07532.1) could form a stable complex with H-2K^b^ following *in vitro* refolding and was selected for crystallization with the H-2K^b^ heavy chain and mβ2m. The peptide–H-2K^b^ (pH-2K^b^) complexes were ultimately concentrated to 10 mg/mL in a buffer containing 20 mM Tris (pH 8.0) and 50 mM NaCl for crystallization. The sample was mixed with reservoir buffer at a 1:1 ratio and crystallized by the sitting-drop vapor diffusion technique at 291 K. An index kit (Hampton Research, Riverside, CA) was used to screen for optimal crystal growth conditions. After several days, crystals of H-2K^b^ complexed with the SVF9 peptide and mβ2m were obtained with Index64 solution [12% (wt/vol) polyethylene glycol 3350, 0.05 M cobalt(II) chloride hexahydrate, 0.05 M nickel(II) chloride hexahydrate, 0.05 M cadmium chloride hydrate, 0.1 M HEPES]. Diffraction data were collected at a resolution of 2.3 Å at the Shanghai Synchrotron Radiation Facility (SSRF) (Shanghai, People’s Republic of China) using Beamline BL18U at a wavelength of 1.5418 Å (69). The crystals were first soaked in reservoir solution containing 25% glycerol as a cryoprotectant and then flash-cooled in a stream of gaseous nitrogen at 100 K. The collected intensities were indexed, integrated, corrected for absorption, scaled, and merged using the HKL3000 package (70).

### Structure determination and refinement.

The crystal of pH-2K^b^ belongs to the P1211 space group, and the structure was solved by molecular replacement using Molrep and Phaser in the CCP4 package, using the mouse H-2K^b^ structure (Protein Data Bank [PDB] accession no. 1VAC) as the search model (71–73). Extensive model building was performed manually with Coot (74), and restrained refinement was conducted using REFMAC5 (75). Additional rounds of refinement were carried out using the Phenix refine program implemented in the Phenix package together with isotropic atomic displacement parameter refinement and bulk solvent modeling (76). The stereochemical quality of the final model was assessed with the PROCHECK program (77).

### Data availability.

The crystal structures have been deposited in the Protein Data Bank (https://www.rcsb.org) under accession no. 7WCY.

## References

[1] Feng Y, Ryan UM, Xiao L. 2018. Genetic diversity and population structure of Cryptosporidium. Trends Parasitol 34:997–1011. doi:10.1016/j.pt.2018.07.009. 30108020

[2] Checkley W, White AC, Jr, Jaganath D, Arrowood MJ, Chalmers RM, Chen X-M, Fayer R, Griffiths JK, Guerrant RL, Hedstrom L, Huston CD, Kotloff KL, Kang G, Mead JR, Miller M, Petri WA, Jr, Priest JW, Roos DS, Striepen B, Thompson RCA, Ward HD, Van Voorhis WA, Xiao L, Zhu G, Houpt ER. 2015. A review of the global burden, novel diagnostics, therapeutics, and vaccine targets for cryptosporidium. Lancet Infect Dis 15:85–94. doi:10.1016/S1473-3099(14)70772-8. 25278220 PMC4401121

[3] Shimelis T, Tassachew Y, Lambiyo T. 2016. Cryptosporidium and other intestinal parasitic infections among HIV patients in southern Ethiopia: significance of improved HIV-related care. Parasit Vectors 9:270. doi:10.1186/s13071-016-1554-x. 27165271 PMC4862162

[4] Vinayak S, Pawlowic MC, Sateriale A, Brooks CF, Studstill CJ, Bar-Peled Y, Cipriano MJ, Striepen B. 2015. Genetic modification of the diarrhoeal pathogen Cryptosporidium parvum. Nature 523:477–480. doi:10.1038/nature14651. 26176919 PMC4640681

[5] Striepen B. 2013. Parasitic infections: time to tackle cryptosporidiosis. Nature 503:189–191. doi:10.1038/503189a. 24236315

[6] Ashigbie PG, Shepherd S, Steiner KL, Amadi B, Aziz N, Manjunatha UH, Spector JM, Diagana TT, Kelly P. 2021. Use-case scenarios for an anti-Cryptosporidium therapeutic. PLoS Negl Trop Dis 15:e0009057. doi:10.1371/journal.pntd.0009057. 33705395 PMC7951839

[7] Manjunatha UH, Vinayak S, Zambriski JA, Chao AT, Sy T, Noble CG, Bonamy GMC, Kondreddi RR, Zou B, Gedeck P, Brooks CF, Herbert GT, Sateriale A, Tandel J, Noh S, Lakshminarayana SB, Lim SH, Goodman LB, Bodenreider C, Feng G, Zhang L, Blasco F, Wagner J, Leong FJ, Striepen B, Diagana TT. 2017. A Cryptosporidium PI(4)K inhibitor is a drug candidate for cryptosporidiosis. Nature 546:376–380. doi:10.1038/nature22337. 28562588 PMC5473467

[8] Huston CD, Spangenberg T, Burrows J, Willis P, Wells TN, van Voorhis W. 2015. A proposed target product profile and developmental cascade for new cryptosporidiosis treatments. PLoS Negl Trop Dis 9:e0003987. doi:10.1371/journal.pntd.0003987. 26447884 PMC4598153

[9] Abubakar I, Aliyu SH, Arumugam C, Usman NK, Hunter PR. 2007. Treatment of cryptosporidiosis in immunocompromised individuals: systematic review and meta-analysis. Br J Clin Pharmacol 63:387–393. doi:10.1111/j.1365-2125.2007.02873.x. 17335543 PMC2203234

[10] Innes EA, Chalmers RM, Wells B, Pawlowic MC. 2020. A One Health approach to tackle cryptosporidiosis. Trends Parasitol 36:290–303. doi:10.1016/j.pt.2019.12.016. 31983609 PMC7106497

[11] Sateriale A, Slapeta J, Baptista R, Engiles JB, Gullicksrud JA, Herbert GT, Brooks CF, Kugler EM, Kissinger JC, Hunter CA, Striepen B. 2019. A genetically tractable, natural mouse model of cryptosporidiosis offers insights into host protective immunity. Cell Host Microbe 26:135–146.e5. doi:10.1016/j.chom.2019.05.006. 31231045 PMC6617386

[12] Mead JR. 2014. Prospects for immunotherapy and vaccines against Cryptosporidium. Hum Vaccin Immunother 10:1505–1513. doi:10.4161/hv.28485. 24638018 PMC4185963

[13] Theodos CM. 1998. Innate and cell-mediated immune responses to Cryptosporidium parvum. Adv Parasitol 40:87–119. doi:10.1016/s0065-308x(08)60118-9. 9554071

[14] Waters WR, Harp JA. 1996. Cryptosporidium parvum infection in T-cell receptor (TCR)-α- and TCR-δ-deficient mice. Infect Immun 64:1854–1857. doi:10.1128/iai.64.5.1854-1857.1996. 8613403 PMC174004

[15] Abrahamsen MS, Lancto CA, Walcheck B, Layton W, Jutila MA. 1997. Localization of α/β and γ/δ T lymphocytes in Cryptosporidium parvum-infected tissues in naive and immune calves. Infect Immun 65:2428–2433. doi:10.1128/iai.65.6.2428-2433.1997. 9169784 PMC175336

[16] Pantenburg B, Castellanos-Gonzalez A, Dann SM, Connelly RL, Lewis DE, Ward HD, White AC, Jr. 2010. Human CD8(+) T cells clear Cryptosporidium parvum from infected intestinal epithelial cells. Am J Trop Med Hyg 82:600–607. doi:10.4269/ajtmh.2010.09-0590. 20348507 PMC2844566

[17] Kváč M, Kodádková A, Sak B, Květoňová D, Jalovecká M, Rost M, Salát J. 2011. Activated CD8+ T cells contribute to clearance of gastric Cryptosporidium muris infections. Parasite Immunol 33:210–216. doi:10.1111/j.1365-3024.2010.01271.x. 21204850

[18] Aguirre SA, Mason PH, Perryman LE. 1994. Susceptibility of major histocompatibility complex (MHC) class I- and MHC class II-deficient mice to Cryptosporidium parvum infection. Infect Immun 62:697–699. doi:10.1128/iai.62.2.697-699.1994. 7905464 PMC186160

[19] Blackwell JM, Roberts CW, Alexander J. 1993. Influence of genes within the MHC on mortality and brain cyst development in mice infected with Toxoplasma gondii: kinetics of immune regulation in BALB H-2 congenic mice. Parasite Immunol 15:317–324. doi:10.1111/j.1365-3024.1993.tb00616.x. 8361774

[20] Madden DR. 1995. The three-dimensional structure of peptide-MHC complexes. Annu Rev Immunol 13:587–622. doi:10.1146/annurev.iy.13.040195.003103. 7612235

[21] Macdonald IK, Harkiolaki M, Hunt L, Connelley T, Carroll AV, MacHugh ND, Graham SP, Jones EY, Morrison WI, Flower DR, Ellis SA. 2010. MHC class I bound to an immunodominant Theileria parva epitope demonstrates unconventional presentation to T cell receptors. PLoS Pathog 6:e1001149. doi:10.1371/journal.ppat.1001149. 20976198 PMC2954893

[22] Feliu V, Vasseur V, Grover HS, Chu HH, Brown MJ, Wang J, Boyle JP, Robey EA, Shastri N, Blanchard N. 2013. Location of the CD8 T cell epitope within the antigenic precursor determines immunogenicity and protection against the Toxoplasma gondii parasite. PLoS Pathog 9:e1003449. doi:10.1371/journal.ppat.1003449. 23818852 PMC3688528

[23] Gibbins MP, Muller K, Glover M, Liu J, Putrianti ED, Bauza K, Reyes-Sandoval A, Matuschewski K, Silvie O, Hafalla JCR. 2020. Importance of the immunodominant CD8^+^ T cell epitope of Plasmodium berghei circumsporozoite protein in parasite- and vaccine-induced protection. Infect Immun 88:e00383-20. doi:10.1128/IAI.00383-20. 32719159 PMC7504970

[24] Pichugin A, Zarling S, Perazzo L, Duffy PE, Ploegh HL, Krzych U. 2018. Identification of a novel CD8 T cell epitope derived from Plasmodium berghei protective liver-stage antigen. Front Immunol 9:91. doi:10.3389/fimmu.2018.00091. 29434602 PMC5796907

[25] Rosenberg CS, Martin DL, Tarleton RL. 2010. CD8+ T cells specific for immunodominant trans-sialidase epitopes contribute to control of Trypanosoma cruzi infection but are not required for resistance. J Immunol 185:560–568. doi:10.4049/jimmunol.1000432. 20530265 PMC3784248

[26] Malik A, Houghten R, Corradin G, Buus S, Berzofsky JA, Hoffman SL. 1995. Identification of a nonameric H-2K^k^-restricted CD8^+^ cytotoxic T lymphocyte epitope on the Plasmodium falciparum circumsporozoite protein. Infect Immun 63:1955–1959. doi:10.1128/iai.63.5.1955-1959.1995. 7537251 PMC173249

[27] Boulter-Bitzer JI, Lee H, Trevors JT. 2007. Molecular targets for detection and immunotherapy in Cryptosporidium parvum. Biotechnol Adv 25:13–44. doi:10.1016/j.biotechadv.2006.08.003. 17055210

[28] Thompson RC, Olson ME, Zhu G, Enomoto S, Abrahamsen MS, Hijjawi NS. 2005. Cryptosporidium and cryptosporidiosis. Adv Parasitol 59:77–158. doi:10.1016/S0065-308X(05)59002-X. 16182865

[29] Toes RE, Hoeben RC, van der Voort EI, Ressing ME, van der Eb AJ, Melief CJ, Offringa R. 1997. Protective anti-tumor immunity induced by vaccination with recombinant adenoviruses encoding multiple tumor-associated cytotoxic T lymphocyte epitopes in a string-of-beads fashion. Proc Natl Acad Sci USA 94:14660–14665. doi:10.1073/pnas.94.26.14660. 9405669 PMC25085

[30] Schulz M, Zinkernagel RM, Hengartner H. 1991. Peptide-induced antiviral protection by cytotoxic T cells. Proc Natl Acad Sci USA 88:991–993. doi:10.1073/pnas.88.3.991. 1992491 PMC50940

[31] Renia L, Marussig MS, Grillot D, Pied S, Corradin G, Miltgen F, Del Giudice G, Mazier D. 1991. In vitro activity of CD4+ and CD8+ T lymphocytes from mice immunized with a synthetic malaria peptide. Proc Natl Acad Sci USA 88:7963–7967. doi:10.1073/pnas.88.18.7963. 1680235 PMC52425

[32] Jurtz V, Paul S, Andreatta M, Marcatili P, Peters B, Nielsen M. 2017. NetMHCpan-4.0: improved peptide-MHC class I interaction predictions integrating eluted ligand and peptide binding affinity data. J Immunol 199:3360–3368. doi:10.4049/jimmunol.1700893. 28978689 PMC5679736

[33] Ellis JM, Henson V, Slack R, Ng J, Hartzman RJ, Katovich Hurley C. 2000. Frequencies of HLA-A2 alleles in five U.S. population groups. Predominance of A*02011 and identification of HLA-A*0231. Hum Immunol 61:334–340. doi:10.1016/s0198-8859(99)00155-x. 10689125

[34] Gonzalez-Galarza FF, Christmas S, Middleton D, Jones AR. 2011. Allele Frequency Net: a database and online repository for immune gene frequencies in worldwide populations. Nucleic Acids Res 39:D913–D919. doi:10.1093/nar/gkq1128. 21062830 PMC3013710

[35] Illing PT, Pymm P, Croft NP, Hilton HG, Jojic V, Han AS, Mendoza JL, Mifsud NA, Dudek NL, McCluskey J, Parham P, Rossjohn J, Vivian JP, Purcell AW. 2018. HLA-B57 micropolymorphism defines the sequence and conformational breadth of the immunopeptidome. Nat Commun 9:4693. doi:10.1038/s41467-018-07109-w. 30410026 PMC6224591

[36] Emini EA, Jameson BA, Wimmer E. 1983. Priming for and induction of anti-poliovirus neutralizing antibodies by synthetic peptides. Nature 304:699–703. doi:10.1038/304699a0. 6310403

[37] Du L, Zhao G, Lin Y, Chan C, He Y, Jiang S, Wu C, Jin DY, Yuen KY, Zhou Y, Zheng BJ. 2008. Priming with rAAV encoding RBD of SARS-CoV S protein and boosting with RBD-specific peptides for T cell epitopes elevated humoral and cellular immune responses against SARS-CoV infection. Vaccine 26:1644–1651. doi:10.1016/j.vaccine.2008.01.025. 18289745 PMC2600875

[38] Carvalho LH, Hafalla JC, Zavala F. 2001. ELISPOT assay to measure antigen-specific murine CD8(+) T cell responses. J Immunol Methods 252:207–218. doi:10.1016/s0022-1759(01)00331-3. 11334981

[39] Fremont DH, Matsumura M, Stura EA, Peterson PA, Wilson IA. 1992. Crystal structures of two viral peptides in complex with murine MHC class I H-2K^b^. Science 257:919–927. doi:10.1126/science.1323877. 1323877

[40] Salti SM, Hammelev EM, Grewal JL, Reddy ST, Zemple SJ, Grossman WJ, Grayson MH, Verbsky JW. 2011. Granzyme B regulates antiviral CD8+ T cell responses. J Immunol 187:6301–6309. doi:10.4049/jimmunol.1100891. 22084442 PMC3237805

[41] Szeto C, Nguyen AT, Lobos CA, Chatzileontiadou DSM, Jayasinghe D, Grant EJ, Riboldi-Tunnicliffe A, Smith C, Gras S. 2021. Molecular basis of a dominant SARS-CoV-2 spike-derived epitope presented by HLA-A*02:01 recognised by a public TCR. Cells 10:2646. doi:10.3390/cells10102646. 34685626 PMC8534114

[42] Meijers R, Lai CC, Yang Y, Liu JH, Zhong W, Wang JH, Reinherz EL. 2005. Crystal structures of murine MHC class I H-2 D(b) and K(b) molecules in complex with CTL epitopes from influenza A virus: implications for TCR repertoire selection and immunodominance. J Mol Biol 345:1099–1110. doi:10.1016/j.jmb.2004.11.023. 15644207

[43] Fremont DH, Stura EA, Matsumura M, Peterson PA, Wilson IA. 1995. Crystal structure of an H-2Kb-ovalbumin peptide complex reveals the interplay of primary and secondary anchor positions in the major histocompatibility complex binding groove. Proc Natl Acad Sci USA 92:2479–2483. doi:10.1073/pnas.92.7.2479. 7708669 PMC42241

[44] Sateriale A, Gullicksrud JA, Engiles JB, McLeod BI, Kugler EM, Henao-Mejia J, Zhou T, Ring AM, Brodsky IE, Hunter CA, Striepen B. 2021. The intestinal parasite Cryptosporidium is controlled by an enterocyte intrinsic inflammasome that depends on NLRP6. Proc Natl Acad Sci USA 118:e2007807118. doi:10.1073/pnas.2007807118. 33372132 PMC7812745

[45] Acosta Rodriguez EV, Araujo Furlan CL, Fiocca Vernengo F, Montes CL, Gruppi A. 2019. Understanding CD8(+) T cell immunity to Trypanosoma cruzi and how to improve it. Trends Parasitol 35:899–917. doi:10.1016/j.pt.2019.08.006. 31607632 PMC6815727

[46] Chu HH, Chan SW, Gosling JP, Blanchard N, Tsitsiklis A, Lythe G, Shastri N, Molina-Paris C, Robey EA. 2016. Continuous effector CD8(+) T cell production in a controlled persistent infection is sustained by a proliferative intermediate population. Immunity 45:159–171. doi:10.1016/j.immuni.2016.06.013. 27421704 PMC4956557

[47] Tosini F, Ludovisi A, Tonanzi D, Amati M, Cherchi S, Pozio E, Gomez-Morales MA. 2019. Delivery of SA35 and SA40 peptides in mice enhances humoral and cellular immune responses and confers protection against Cryptosporidium parvum infection. Parasit Vectors 12:233. doi:10.1186/s13071-019-3486-8. 31092283 PMC6518611

[48] Bartelt LA, Bolick DT, Kolling GL, Roche JK, Zaenker EI, Lara AM, Noronha FJ, Cowardin CA, Moore JH, Turner JR, Warren CA, Buck GA, Guerrant RL. 2016. Cryptosporidium priming is more effective than vaccine for protection against cryptosporidiosis in a murine protein malnutrition model. PLoS Negl Trop Dis 10:e0004820. doi:10.1371/journal.pntd.0004820. 27467505 PMC4965189

[49] Ivanova DL, Denton SL, Fettel KD, Sondgeroth KS, Munoz Gutierrez J, Bangoura B, Dunay IR, Gigley JP. 2019. Innate lymphoid cells in protection, pathology, and adaptive immunity during apicomplexan infection. Front Immunol 10:196. doi:10.3389/fimmu.2019.00196. 30873151 PMC6403415

[50] Aboelsoued D, Toaleb NI, Abdel Megeed KN, Hassan SE, Ibrahim S. 2019. Cellular immune response and scanning electron microscopy in the evaluation of Moringa leaves aqueous extract effect on Cryptosporidium parvum in buffalo intestinal tissue explants. J Parasit Dis 43:393–401. doi:10.1007/s12639-019-01103-9. 31406404 PMC6667529

[51] Tessema TS, Schwamb B, Lochner M, Forster I, Jakobi V, Petry F. 2009. Dynamics of gut mucosal and systemic Th1/Th2 cytokine responses in interferon-gamma and interleukin-12p40 knock out mice during primary and challenge Cryptosporidium parvum infection. Immunobiology 214:454–466. doi:10.1016/j.imbio.2008.11.015. 19155092

[52] Barakat FM, McDonald V, Di Santo JP, Korbel DS. 2009. Roles for NK cells and an NK cell-independent source of intestinal gamma interferon for innate immunity to Cryptosporidium parvum infection. Infect Immun 77:5044–5049. doi:10.1128/IAI.00377-09. 19687195 PMC2772539

[53] Bedi B, McNair NN, Mead JR. 2014. Dendritic cells play a role in host susceptibility to Cryptosporidium parvum infection. Immunol Lett 158:42–51. doi:10.1016/j.imlet.2013.11.015. 24295591

[54] Bijker MS, van den Eeden SJ, Franken KL, Melief CJ, Offringa R, van der Burg SH. 2007. CD8+ CTL priming by exact peptide epitopes in incomplete Freund’s adjuvant induces a vanishing CTL response, whereas long peptides induce sustained CTL reactivity. J Immunol 179:5033–5040. doi:10.4049/jimmunol.179.8.5033. 17911588

[55] Xiao L, Feng Y. 2017. Molecular epidemiologic tools for waterborne pathogens Cryptosporidium spp. and Giardia duodenalis. Food Waterborne Parasitol 8–9:14–32. doi:10.1016/j.fawpar.2017.09.002. PMC7034008 32095639

[56] Garcia KC, Adams JJ, Feng D, Ely LK. 2009. The molecular basis of TCR germline bias for MHC is surprisingly simple. Nat Immunol 10:143–147. doi:10.1038/ni.f.219. 19148199 PMC3982143

[57] Lo Conte L, Chothia C, Janin J. 1999. The atomic structure of protein-protein recognition sites. J Mol Biol 285:2177–2198. doi:10.1006/jmbi.1998.2439. 9925793

[58] Kuhns JJ, Batalia MA, Yan S, Collins EJ. 1999. Poor binding of a HER-2/neu epitope (GP2) to HLA-A2.1 is due to a lack of interactions with the center of the peptide. J Biol Chem 274:36422–36427. doi:10.1074/jbc.274.51.36422. 10593938

[59] Xu R, Feng Y, Xiao L, Sibley LD. 2021. Insulinase-like protease 1 contributes to macrogamont formation in Cryptosporidium parvum. mBio 12:e03405-20. doi:10.1128/mBio.03405-20. 33688009 PMC8092296

[60] Dumaine JE, Sateriale A, Gibson AR, Reddy AG, Gullicksrud JA, Hunter EN, Clark JT, Striepen B. 2021. The enteric pathogen Cryptosporidium parvum exports proteins into the cytosol of the infected host cell. Elife 10:e70451. doi:10.7554/eLife.70451. 34866573 PMC8687662

[61] Guerin A, Roy NH, Kugler EM, Berry L, Burkhardt JK, Shin JB, Striepen B. 2021. Cryptosporidium rhoptry effector protein ROP1 injected during invasion targets the host cytoskeletal modulator LMO7. Cell Host Microbe 29:1407–1420.e5. doi:10.1016/j.chom.2021.07.002. 34348092 PMC8475647

[62] Ogden BE, Pang William W, Agui T, Lee BH. 2016. Laboratory animal laws, regulations, guidelines and standards in China Mainland, Japan, and Korea. ILAR J 57:301–311. 29117401 10.1093/ilar/ilw018

[63] Fan S, Wu Y, Wang S, Wang Z, Jiang B, Liu Y, Liang R, Zhou W, Zhang N, Xia C. 2016. Structural and biochemical analyses of swine major histocompatibility complex class I complexes and prediction of the epitope map of important influenza A virus strains. J Virol 90:6625–6641. doi:10.1128/JVI.00119-16. 27170754 PMC4944273

[64] Zhou M, Xu Y, Lou Z, Cole DK, Li X, Liu Y, Tien P, Rao Z, Gao GF. 2004. Complex assembly, crystallization and preliminary X-ray crystallographic studies of MHC H-2K^d^ complexed with an HBV-core nonapeptide. Acta Crystallogr D Biol Crystallogr 60:1473–1475. doi:10.1107/S0907444904013587. 15272181

[65] Ma R-X, Cheng L-F, Ying Q-K, Liu R-R, Ma T-J, Zhang X-X, Liu Z-Y, Zhang L, Ye W, Zhang F-L, Xu Z-K, Wang F, Wu X-A. 2016. Screening and identification of an H-2K^b^-restricted CTL epitope within the glycoprotein of Hantaan virus. Front Cell Infect Microbiol 6:151. doi:10.3389/fcimb.2016.00151. 27933274 PMC5122572

[66] Weigmann B, Tubbe I, Seidel D, Nicolaev A, Becker C, Neurath MF. 2007. Isolation and subsequent analysis of murine lamina propria mononuclear cells from colonic tissue. Nat Protoc 2:2307–2311. doi:10.1038/nprot.2007.315. 17947970

[67] Wang L, Cao L, Zheng S, Chang Y, Zhang K, Zhang S, Zhang L. 2021. Molecular identification and biological characterization of Cryptosporidium muris from camels (Camelus bactrianus) in China. Parasit Vectors 14:365. doi:10.1186/s13071-021-04862-8. 34266490 PMC8281508

[68] Arrowood MJ, Sterling CR. 1987. Isolation of Cryptosporidium oocysts and sporozoites using discontinuous sucrose and isopycnic Percoll gradients. J Parasitol 73:314–319. doi:10.2307/3282084. 3585626

[69] Zhang W-Z, Tang J-C, Wang S-S, Wang Z-J, Qin W-M, He J-H. 2019. The protein complex crystallography beamline (BL19U1) at the Shanghai Synchrotron Radiation Facility. Nucl Sci Tech 30:170. doi:10.1007/s41365-019-0683-2.

[70] Otwinowski Z, Minor W. 1997. Processing of X-ray diffraction data collected in oscillation mode. Methods Enzymol 276:307–326. doi:10.1016/S0076-6879(97)76066-X. 27754618

[71] Lebedev AA, Vagin AA, Murshudov GN. 2008. Model preparation in MOLREP and examples of model improvement using X-ray data. Acta Crystallogr D Biol Crystallogr 64:33–39. doi:10.1107/S0907444907049839. 18094465 PMC2394799

[72] McCoy AJ. 2007. Solving structures of protein complexes by molecular replacement with Phaser. Acta Crystallogr D Biol Crystallogr 63:32–41. doi:10.1107/S0907444906045975. 17164524 PMC2483468

[73] Collaborative Computational Project, Number 4. 1994. The CCP4 suite: programs for protein crystallography. Acta Crystallogr D Biol Crystallogr 50:760–763. doi:10.1107/S0907444994003112. 15299374

[74] Emsley P, Cowtan K. 2004. Coot: model-building tools for molecular graphics. Acta Crystallogr D Biol Crystallogr 60:2126–2132. doi:10.1107/S0907444904019158. 15572765

[75] Murshudov GN, Vagin AA, Dodson EJ. 1997. Refinement of macromolecular structures by the maximum-likelihood method. Acta Crystallogr D Biol Crystallogr 53:240–255. doi:10.1107/S0907444996012255. 15299926

[76] Adams PD, Grosse-Kunstleve RW, Hung LW, Ioerger TR, McCoy AJ, Moriarty NW, Read RJ, Sacchettini JC, Sauter NK, Terwilliger TC. 2002. PHENIX: building new software for automated crystallographic structure determination. Acta Crystallogr D Biol Crystallogr 58:1948–1954. doi:10.1107/s0907444902016657. 12393927

[77] Laskowski RA, Moss DS, Thornton JM. 1993. Main-chain bond lengths and bond angles in protein structures. J Mol Biol 231:1049–1067. doi:10.1006/jmbi.1993.1351. 8515464

