# Supplementary material for: Structural Analyses of a Dominant Cryptosporidium parvum Epitope Presented by H-2Kb Offer New Options To Combat Cryptosporidiosis
Source: mBio. 2023 Jan 5;14(1):e02666-22. doi: 10.1128/mbio.02666-22 (PMC9973275; doi:10.1128/mbio.02666-22)
Supplement: TABLE S1 [file mbio.02666-22-s0004.docx]

**Table S1. Geographic distributions of HLA-A*0201 and worldwide distribution of *C. parvum* outbreaks and morbidity/mortality.**

| Population/country | % of individuals that have the allele | Allele Frequency  (in decimals) | *Cryptosporidium parvum* that have been reported |
| --- | --- | --- | --- |
| Bolivia/peru Quechua | 85.7 | 0.6667 | (1-7) |
| Canada Chipewyan | 76.0 | 0.4800 | (8-10) |
| Paraguay/Argentina | 70.0 | 0.3500 | (11-19) |
| Russia Tundra Nentsi | 62.5 | 0.3750 | (20) |
| Mexico Zapotec | 55 | 0.4000 | (21-26) |
| England | 50.5 | 0.3035 | (27-32) |
| United States | 49.6 | 0.29 | (33-35) |
| Poland BMR | 49.3 | 0.2848 | (36-42) |
| USA caucasian | 47.0 | 0.2747 | (43-45) |
| Australia | 46.5 | 0.2680 | (46, 47) |
| Spain, Canary Islands | 40.5 | 0.2372 | (48-53) |
| Nigeria | 31.4 | 0.1783 | (54) |
| Kenya | 27.0 | 0.1450 | (55) |
| India | 13.8 | 0.0730 | (56-58) |
| China Hong kong | 12.7 | 0.0653 | (59) |
| China Guangdong |  | 0.0050 | (60-62) |

1. Gomez-Puerta LA, Gonzalez AE, Vargas-Calla A, Lopez-Urbina MT, Cama V, Xiao L. 2020. Cryptosporidium parvum as a risk factor of diarrhea occurrence in neonatal alpacas in Peru. Parasitol Res 119:243-248.

2. Gomez-Couso H, Ortega-Mora LM, Aguado-Martinez A, Rosadio-Alcantara R, Maturrano-Hernandez L, Luna-Espinoza L, Zanabria-Huisa V, Pedraza-Diaz S. 2012. Presence and molecular characterisation of Giardia and Cryptosporidium in alpacas (Vicugna pacos) from Peru. Vet Parasitol 187:414-20.

3. Xiao L, Bern C, Limor J, Sulaiman I, Roberts J, Checkley W, Cabrera L, Gilman RH, Lal AA. 2001. Identification of 5 types of Cryptosporidium parasites in children in Lima, Peru. J Infect Dis 183:492-7.

4. Zhang Y, Lee B, Thompson M, Glass R, Cama RI, Figueroa D, Gilman R, Taylor D, Stephenson C. 2000. Lactulose-mannitol intestinal permeability test in children with diarrhea caused by rotavirus and cryptosporidium. Diarrhea Working Group, Peru. J Pediatr Gastroenterol Nutr 31:16-21.

5. Checkley W, Epstein LD, Gilman RH, Black RE, Cabrera L, Sterling CR. 1998. Effects of Cryptosporidium parvum infection in Peruvian children: growth faltering and subsequent catch-up growth. Am J Epidemiol 148:497-506.

6. Ortega YR, Roxas CR, Gilman RH, Miller NJ, Cabrera L, Taquiri C, Sterling CR. 1997. Isolation of Cryptosporidium parvum and Cyclospora cayetanensis from vegetables collected in markets of an endemic region in Peru. Am J Trop Med Hyg 57:683-6.

7. Checkley W, Gilman RH, Epstein LD, Suarez M, Diaz JF, Cabrera L, Black RE, Sterling CR. 1997. Asymptomatic and symptomatic cryptosporidiosis: their acute effect on weight gain in Peruvian children. Am J Epidemiol 145:156-63.

8. Ayres Hutter J, Dion R, Irace-Cima A, Fiset M, Guy R, Dixon B, Aguilar JL, Trepanier J, Thivierge K. 2020. Cryptosporidium spp.: Human incidence, molecular characterization and associated exposures in Quebec, Canada (2016-2017). PLoS One 15:e0228986.

9. Budu-Amoako E, Greenwood SJ, Dixon BR, Barkema HW, McClure JT. 2012. Occurrence of Cryptosporidium and Giardia on beef farms and water sources within the vicinity of the farms on Prince Edward Island, Canada. Vet Parasitol 184:1-9.

10. Coklin T, Farber J, Parrington L, Dixon B. 2007. Prevalence and molecular characterization of Giardia duodenalis and Cryptosporidium spp. in dairy cattle in Ontario, Canada. Vet Parasitol 150:297-305.

11. Garro CJ, Morici GE, Tomazic ML, Vilte D, Encinas M, Vega C, Bok M, Parreno V, Schnittger L. 2021. Occurrence of Cryptosporidium and other enteropathogens and their association with diarrhea in dairy calves of Buenos Aires province, Argentina. Vet Parasitol Reg Stud Reports 24:100567.

12. Schares G, Dubey JP, Rosenthal B, Tuschy M, Barwald A, Conraths FJ. 2020. Sensitive, quantitative detection of Besnoitia darlingi and related parasites in intermediate hosts and to assess felids as definitive hosts for known and as-yet undescribed related parasite species. Int J Parasitol Parasites Wildl 11:114-119.

13. Lombardelli JA, Tomazic ML, Schnittger L, Tiranti KI. 2019. Prevalence of Cryptosporidium parvum in dairy calves and GP60 subtyping of diarrheic calves in central Argentina. Parasitol Res 118:2079-2086.

14. Peralta RH, Velasquez JN, Cunha Fde S, Pantano ML, Sodre FC, Silva S, Astudillo OG, Peralta JM, Carnevale S. 2016. Genetic diversity of Cryptosporidium identified in clinical samples from cities in Brazil and Argentina. Mem Inst Oswaldo Cruz 111:30-6.

15. Cimino RO, Jeun R, Juarez M, Cajal PS, Vargas P, Echazu A, Bryan PE, Nasser J, Krolewiecki A, Mejia R. 2015. Identification of human intestinal parasites affecting an asymptomatic peri-urban Argentinian population using multi-parallel quantitative real-time polymerase chain reaction. Parasit Vectors 8:380.

16. Del Coco VF, Cordoba MA, Bilbao G, de Almeida Castro AP, Basualdo JA, Fayer R, Santin M. 2014. Cryptosporidium parvum GP60 subtypes in dairy cattle from Buenos Aires, Argentina. Res Vet Sci 96:311-4.

17. Tomazic ML, Maidana J, Dominguez M, Uriarte EL, Galarza R, Garro C, Florin-Christensen M, Schnittger L. 2013. Molecular characterization of Cryptosporidium isolates from calves in Argentina. Vet Parasitol 198:382-6.

18. Netor Velasquez J, Marta E, Alicia di Risio C, Etchart C, Gancedo E, Victor Chertcoff A, Bruno Malandrini J, German Astudillo O, Carnevale S. 2012. Molecular identification of protozoa causing AIDS-associated cholangiopathy in Buenos Aires, Argentina. Acta Gastroenterol Latinoam 42:301-8.

19. Barboni G, Candi M, Ines Villace M, Leonardelli A, Balbaryski J, Gaddi E. 2008. [Intestinal cryptosporidiosis in HIV infected children]. Medicina (B Aires) 68:213-8.

20. Kucerova Z, Sokolova OI, Demyanov AV, Kvac M, Sak B, Kvetonova D, Secor WE. 2011. Microsporidiosis and Cryptosporidiosis in HIV/AIDS Patients in St. Petersburg, Russia: Serological identification of microsporidia and Cryptosporidium parvum in sera samples from HIV/AIDS patients. AIDS Res Hum Retroviruses 27:13-5.

21. Morrow JJ, Reinhard KJ. 2016. Cryptosporidium parvum Among Coprolites from La Cueva de los Muertos Chiquitos (600-800 CE), Rio Zape Valley, Durango, Mexico. J Parasitol 102:429-35.

22. Nair P, Mohamed JA, DuPont HL, Figueroa JF, Carlin LG, Jiang ZD, Belkind-Gerson J, Martinez-Sandoval FG, Okhuysen PC. 2008. Epidemiology of cryptosporidiosis in North American travelers to Mexico. Am J Trop Med Hyg 79:210-4.

23. Diaz E, Mondragon J, Ramirez E, Bernal R. 2003. Epidemiology and control of intestinal parasites with nitazoxanide in children in Mexico. Am J Trop Med Hyg 68:384-5.

24. Kuhn RC, Rock CM, Oshima KH. 2002. Occurrence of Cryptosporidium and Giardia in wild ducks along the Rio Grande River valley in southern New Mexico. Appl Environ Microbiol 68:161-5.

25. Leach CT, Koo FC, Kuhls TL, Hilsenbeck SG, Jenson HB. 2000. Prevalence of Cryptosporidium parvum infection in children along the Texas-Mexico border and associated risk factors. Am J Trop Med Hyg 62:656-61.

26. Rossignol JF, Hidalgo H, Feregrino M, Higuera F, Gomez WH, Romero JL, Padierna J, Geyne A, Ayers MS. 1998. A double-'blind' placebo-controlled study of nitazoxanide in the treatment of cryptosporidial diarrhoea in AIDS patients in Mexico. Trans R Soc Trop Med Hyg 92:663-6.

27. Utsi L, Smith SJ, Chalmers RM, Padfield S. 2016. Cryptosporidiosis outbreak in visitors of a UK industry-compliant petting farm caused by a rare Cryptosporidium parvum subtype: a case-control study. Epidemiol Infect 144:1000-9.

28. McKerr C, Adak GK, Nichols G, Gorton R, Chalmers RM, Kafatos G, Cosford P, Charlett A, Reacher M, Pollock KG, Alexander CL, Morton S. 2015. An Outbreak of Cryptosporidium parvum across England & Scotland Associated with Consumption of Fresh Pre-Cut Salad Leaves, May 2012. PLoS One 10:e0125955.

29. Hoek MR, Oliver I, Barlow M, Heard L, Chalmers R, Paynter S. 2008. Outbreak of Cryptosporidium parvum among children after a school excursion to an adventure farm, south west England. J Water Health 6:333-8.

30. Pritchard GC, Marshall JA, Giles M, Chalmers RM, Marshall RN. 2007. Cryptosporidium parvum infection in orphan lambs on a farm open to the public. Vet Rec 161:11-4.

31. Hunter PR, Hadfield SJ, Wilkinson D, Lake IR, Harrison FC, Chalmers RM. 2007. Subtypes of Cryptosporidium parvum in humans and disease risk. Emerg Infect Dis 13:82-8.

32. Louie K, Gustafson L, Fyfe M, Gill I, MacDougall L, Tom L, Wong Q, Isaac-Renton J. 2004. An outbreak of Cryptosporidium parvum in a Surrey pool with detection in pool water sampling. Can Commun Dis Rep 30:61-6.

33. Becker DJ, Oloya J, Ezeamama AE. 2015. Household Socioeconomic and Demographic Correlates of Cryptosporidium Seropositivity in the United States. PLoS Negl Trop Dis 9:e0004080.

34. Fayer R, Santin M, Dargatz D. 2010. Species of Cryptosporidium detected in weaned cattle on cow-calf operations in the United States. Vet Parasitol 170:187-92.

35. Xiao L, Zhou L, Santin M, Yang W, Fayer R. 2007. Distribution of Cryptosporidium parvum subtypes in calves in eastern United States. Parasitol Res 100:701-6.

36. Piekara-Stepinska A, Piekarska J, Gorczykowski M. 2021. Cryptosporidium spp. in dogs and cats in Poland. Ann Agric Environ Med 28:345-347.

37. Kaupke A, Rzezutka A. 2015. Emergence of novel subtypes of Cryptosporidium parvum in calves in Poland. Parasitol Res 114:4709-16.

38. Perec-Matysiak A, Bunkowska-Gawlik K, Zalesny G, Hildebrand J. 2015. Small rodents as reservoirs of Cryptosporidium spp. and Giardia spp. in south-western Poland. Ann Agric Environ Med 22:1-5.

39. Adamska M. 2015. Molecular characterization of Cryptosporidium and Giardia occurring in natural water bodies in Poland. Parasitol Res 114:687-92.

40. Paziewska A, Bednarska M, Nieweglowski H, Karbowiak G, Bajer A. 2007. Distribution of Cryptosporidium and Giardia spp. in selected species of protected and game mammals from North-Eastern Poland. Ann Agric Environ Med 14:265-70.

41. Bajer A, Caccio S, Bednarska M, Behnke JM, Pieniazek NJ, Sinski E. 2003. Preliminary molecular characterization of Cryptosporidium parvum isolates of wildlife rodents from Poland. J Parasitol 89:1053-5.

42. Sinski E. 1993. Cryptosporidiosis in Poland: clinical, epidemiologic and parasitologic aspects. Folia Parasitol (Praha) 40:297-300.

43. Hussain S, Mohsin Bukhari S, Wang L, Khalid N, Hou Z. 2021. Exploration of Zoo felids in North-East China for the prevalence and molecular identification of Cryptosporidium spp. PeerJ 9:e11819.

44. Li X, Atwill ER. 2021. Diverse Genotypes and Species of Cryptosporidium in Wild Rodent Species from the West Coast of the USA and Implications for Raw Produce Safety and Microbial Water Quality. Microorganisms 9.

45. Marquis ND, Bishop TJ, Record NR, Countway PD, Fernandez Robledo JA. 2019. Molecular Epizootiology of Toxoplasma gondii and Cryptosporidium parvum in the Eastern Oyster (Crassostrea virginica) from Maine (USA). Pathogens 8.

46. Kong FE, Deighton MA, Thurbon NA, Smith SR, Rouch DA. 2018. Cryptosporidium parvum decay during air drying and stockpiling of mesophilic anaerobically digested sewage sludge in a simulation experiment and oocyst counts in sludge collected from operational treatment lagoons in Victoria, Australia. J Water Health 16:435-448.

47. Zahedi A, Lee GKC, Greay TL, Walsh AL, Blignaut DJC, Ryan UM. 2018. First report of Cryptosporidium parvum in a dromedary camel calf from Western Australia. Acta Parasitol 63:422-427.

48. Ramo A, Quilez J, Monteagudo L, Del Cacho E, Sanchez-Acedo C. 2016. Intra-Species Diversity and Panmictic Structure of Cryptosporidium parvum Populations in Cattle Farms in Northern Spain. PLoS One 11:e0148811.

49. Quilez J, Vergara-Castiblanco C, Monteagudo L, del Cacho E, Sanchez-Acedo C. 2013. Host association of Cryptosporidium parvum populations infecting domestic ruminants in Spain. Appl Environ Microbiol 79:5363-71.

50. Causape AC, Quilez J, Sanchez-Acedo C, del Cacho E, Lopez-Bernad F. 2002. Prevalence and analysis of potential risk factors for Cryptosporidium parvum infection in lambs in Zaragoza (northeastern Spain). Vet Parasitol 104:287-98.

51. Torres J, Gracenea M, Gomez MS, Arrizabalaga A, Gonzalez-Moreno O. 2000. The occurrence of Cryptosporidium parvum and C. muris in wild rodents and insectivores in Spain. Vet Parasitol 92:253-60.

52. Causape AC, Quilez J, Sanchez-Acedo C, del Cacho E. 1996. Prevalence of intestinal parasites, including Cryptosporidium parvum, in dogs in Zaragoza city, Spain. Vet Parasitol 67:161-7.

53. Villacorta I, Ares-Mazas E, Lorenzo MJ. 1991. Cryptosporidium parvum in cattle, sheep and pigs in Galicia (N.W. Spain). Vet Parasitol 38:249-52.

54. Ayinmode AB, Agbajelola VI. 2019. Molecular identification of Cryptosporidium parvum in rabbits (Oryctolagus cuniculus) in Nigeria. Ann Parasitol 65:237-243.

55. Swierczewski B, Odundo E, Ndonye J, Kirera R, Odhiambo C, Oaks E. 2012. Comparison of the Triage Micro Parasite Panel and Microscopy for the Detection of Entamoeba histolytica/Entamoeba dispar, Giardia lamblia, and Cryptosporidium parvum in Stool Samples Collected in Kenya. J Trop Med 2012:564721.

56. Joute JR, Gill JP, Singh BB. 2016. Prevalence and molecular epidemiology of Cryptosporidium parvum in dairy calves in Punjab (India). J Parasit Dis 40:745-9.

57. Rajkhowa S, Rajkhowa C, Hazarika GC. 2006. Prevalence of Cryptosporidium parvum in mithuns (Bos frontalis) from India. Vet Parasitol 142:146-9.

58. Singh BB, Sharma R, Kumar H, Banga HS, Aulakh RS, Gill JP, Sharma JK. 2006. Prevalence of Cryptosporidium parvum infection in Punjab (India) and its association with diarrhea in neonatal dairy calves. Vet Parasitol 140:162-5.

59. Ho KC, Chow YL, Yau JT. 2003. Chemical and microbiological qualities of The East River (Dongjiang) water, with particular reference to drinking water supply in Hong Kong. Chemosphere 52:1441-50.

60. Chen J, Wang W, Lin Y, Sun L, Li N, Guo Y, Kvac M, Ryan U, Feng Y, Xiao L. 2021. Genetic characterizations of Cryptosporidium spp. from pet rodents indicate high zoonotic potential of pathogens from chinchillas. One Health 13:100269.

61. Li F, Zhao W, Zhang C, Guo Y, Li N, Xiao L, Feng Y. 2020. Cryptosporidium Species and C. parvum Subtypes in Farmed Bamboo Rats. Pathogens 9.

62. Mi R, Wang X, Huang Y, Zhou P, Liu Y, Chen Y, Chen J, Zhu W, Chen Z. 2014. Prevalence and molecular characterization of Cryptosporidium in goats across four provincial level areas in China. PLoS One 9:e111164.
