# Supplementary material for: Structural Analyses of a Dominant Cryptosporidium parvum Epitope Presented by H-2Kb Offer New Options To Combat Cryptosporidiosis
Source: mBio. 2023 Jan 5;14(1):e02666-22. doi: 10.1128/mbio.02666-22 (PMC9973275; doi:10.1128/mbio.02666-22)
Supplement: TABLE S2 [file mbio.02666-22-s0005.doc]

**Table S2. Complete list of the peptides selected from *C. parvum* by *in silico* prediction methods.**

| Allele | H-2K^b^ | | | HLA-A*0201 | | |
| --- | --- | --- | --- | --- | --- | --- |
| Derived protein | Sequence^a^ | IC50 values | BindLevel | Sequence^a^ | IC50 values | BindLevel |
| CSL | ITVKYHAL  IRLAFLRL  IWHKSVNL  SLNPYSLL  SMIWHKSV  ALYDAYCIL  MIWHKSVNL  AYFCRCGGL  RITVKYHAL  EVFAYFCRC  NSMIWHKSV  DSLNPYSLL  FAYFCRCGGL  SMIWHKSVNL  KEVFAYFCRC  VFAYFCRCGGL  NSMIWHKSVNL | 10.15 nM  93.15 nM  313.95 nM  690.73 nM  924.46 nM  128.7nM  190.3nM  388.7 nM  549.0 nM  645.9 nM  913.3 nM  989.7 nM  293.9 nM  319.0 nM  903.5 nM  616.4nM  1030.62nM | SB  SB  WB  WB  WB  SB  SB  WB  WB  WB  WB  WB  WB  WB  WB  WB  WB | ALYDAYCIL  MIWHKSVNL YMDEDGKEV  SLSNYNSMI  SLNPYSLLGV  YDLSAFNESL  SMIWHKSVNL  YHALYDAYCIL | 39.6nM  72.2nM  17.5nM  160.7 nM  15.1 nM  45.0 nM  172.2 nM  298.49nM | WB  WB  SB  WB  SB  WB  WB  WB |
| gp40/15 | YTIVYAPI  VFAIFAAL  SAFGLRYI  KSYNTVKI  AVFSAPAV  SSRSRRSL  SVFAIFAAL  IVPSVFAIF  VSAVFSAPA  AIFAALFVL  VFAIFAALF  SAFGLRYIV  VTFEKSYNT  TVDLFAFTL  VNGQDFSTL  AYTIVYAPI  SVFAIFAALF  VTFEKSYNTV  ATVDLFAFTL  VSAVFSAPAV  AVFSAPAVPL  GAYTIVYAPI  VPSVFAIFAAL  SVFAIFAALFV  PSVFAIFAALF  IVSAVFSAPAV  SVTFEKSYNTV  VSAVFSAPAVP  VTFEKSYNTVK | 14.49 nM  14.53 nM  555.61 nM  908.04 nM  938.87 nM  1179.35 nM  10.2nM  210.5nM  222.6 nM  589.4nM  744.5 nM  754.6 nM  842.1 nM  860.4 nM  886.8 nM  988.7 nM  94.6 nM  297.1nM  366.0 nM  410.3 nM  751.5 nM  906.2 nM  153.99 nM  244.70 nM  263.26nM  618.26nM  627.20nM  788.16nM  808.21nM | SB  SB  WB  WB  WB  WB  SB  WB  WB  WB  WB  WB  WB  WB  WB  WB  SB  WB  WB  WB  WB  WB  SB  WB  WB  WB  WB  WB  WB | AIFAALFV  SVFAIFAAL  YIVPSVFAI  LLSVIVSAV  YISGEVTSV  AIFAALFVL  VLLSVIVSA  SLIIVLLSV  RLSLIIVLL  RLNENGDLV  FAIFAALFV  GLRYIVPSV  SLSEETSEA  VIVSAVFSA  FVMWFGEGT  VLLSVIVSAV  FAIFAALFVL  YRLNENGDLV  IVLLSVIVSA  FGLRYIVPSV  VMWFGEGTPA  MRLSLIIVLL  VTFEKSYNTV  IVLLSVIVSAV | 258.25 nM  103.8nM  7.9nM  15.4nM  17.6nM  29.3nM  29.9nM  42.6 nM  72.6 nM  77.3 nM  84.2 nM  138.1 nM  213.4 nM  216.9 nM  224.5 nM  23.6 nM  62.4 nM  83.8 nM  84.0 nM  109.1 nM  124.9 nM  140.3 nM  699.5nM  182.73 nM | WB  WB  SB  SB  SB  SB  SB  WB  WB  WB  WB  WB  WB  WB  WB  SB  WB  WB  WB  WB  WB  WB  WB  WB |
| Gp900 | VSGVFATV  VVGEFGGL  VSLISAPI  VAVIMNPL  ASVIHTAL  VSITSGEL  SGLSVSYL  ISYLPFAK  GSVPYVSL  HSGTFMPV  IMNPLFSL  SSAYSAPF  IPNTYAGV  MNPLFSLA  VSSSAIAL  IYDYNSGL  SAIALVAV  KKYFAANV  ANTNFLLV  FIPGFNVL  MIYDYNSGL  KMLDKYTRM  VSYLAAKNL  VIMNPLFSL  SAYSAPFEL  SVSGVFATV  LAFKSSNRL  VVGEFGGLL  MNPLFSLAF  TSSAYSAPF  TGLPFIPGF  THVRFRFKV  MVVGEFGGL  TSMNWPVSI  LNFTSTTGF  SGKYFSGSI  SGYQTSADF  VIHHSGTNL  KVYIPYTKC  ISYLPFAKN  TIPNTYAGV  KGSVPYVSL  MIYDYNSGLL  RMIYDYNSGL  ISGKYFSGSI  IMNPLFSLAF  ISYLPFAKNG  AAKKYFAANV  LSVSYLAAKNL  IEKMLDKYTRM  RMIYDYNSGLL  TRMIYDYNSGL  MIYDYNSGLLL  FSLAFKSSNRL  VSYLAAKNLTV  SSFAGAYKYAV  VSNEKFVIPSL  SVSYLAAKNLT  PISGKYFSGSI | 13.47nM  41.83nM  42.49nM  79.18nM  113.41nM  196.52nM  220.91nM  327.25nM  392.03nM  409.07nM  558.35nM  625.12nM  667.11nM  667.11nM  811.11nM  883.86nM  936.13nM  956.42nM  991.35nM  1014.07nM  27.3nM  105.4nM  12.5nM  169.7nM  186.4nM  978.7nM  243.1 nM  265.8 nM  306.9 nM  325.5 nM  360.4 nM  462.3 nM  493.3 nM  498.1 nM  642.3 nM  716.7 nM  735.4 nM  756.4 nM  779.0 nM  816.8 nM  898.4 nM  1022.3 nM  172.0 nM  217.5 nM  465.1 nM  512.4 nM  540.2 nM  771.0 nM  181.39 nM  388.01 nM  472.73 nM  479.62 nM  480.98 nM  657.57 nM  692.59 nM  801.36 nM  959.45 nM  979.16 nM  1157.81 nM | SB  SB  SB  SB  SB  SB  WB  WB  WB  WB  WB  WB  WB  WB  WB  WB  WB  WB  WB  WB  SB  SB  SB  SB  SB  WB  WB  WB  WB  WB  WB  WB  WB  WB  WB  WB  WB  WB  WB  WB  WB  WB  SB  WB  WB  WB  WB  WB  SB  WB  WB  WB  WB  WB  WB  WB  WB  WB  WB | LDFTIPPV  KLSIPPSV  KLLLQLSV  MIYDYNSGL  KMLDKYTRM  VIMNPLFSL  MLFDNSTGV  YLAAKNLTV  SLDFTIPPV  ALVEXLATA  FIPGFNVLV  XMMENTMET  SVSGVFATV  KISGQTFQV  SIAGDLNPI  GLLNPATGV  IMNPLFSLA  SLIPFNPET  IMNGTIAGI  MVDPVSLML  NLFDPSTKL  KLIDPESGI  ILNPIITTT  MLDKYTRMI  SISELMYDI  KLLLQLSVI  VLNITTDEV  LLDSNDEPI  QQMKLLLQL  KMLDKYTRM  XMACCESSI  RMVDPVSLM  ILEGSIAGI  SVPYVSLYV  ITYXFCALI  LLSQKSAPI  TIAGIVSGI  SLMLFDNST  TMDSSFAGA  LMLFDNSTGV  RSLDFTIPPV  ALVAVIMNPL  RMVDPVSLML  GLPIDPMVGL  TLTGYPLDPV  SDLDPTTFLL  GLPFIPGFNV  YIPYTKCVGV  TMSGLSVSYL  AVIMNPLFSL  IMNGTIAGIV  RMIYDYNSGL  FELDVSGVPI  LLLDSNDEPI  FLLVDPKINA  GLPIDLETGL  LMYDIESGRL  QMKLLLQLSV  FVIPSLPSDL  VIMNPLFSLA  SILEGSIAGI  MIYDYNSGLL  GLPVDEITGL  KLPIDGNNQL  ILTEVLNITT  LLNPATGVMI  YLPFAKNGEL  KMLDKYTRMI  SLMLFDNSTGV  DRSLDFTIPPV FTTDTSMNWPV  VSYLAAKNLTV  FGGLLNPATGV  MIYDYNSGLLL  QIADTSNLFPV | 122.42nM  172.78nM  192.95nM  15.5nM  171.1nM  20.6nM  2.9nM  5.4nM  6.7nM  8.5nM  15.3nM  30.9nM  34.2nM  38.2nM  38.5 nM  44.5 nM  48.2 nM  52.1 nM  52.3 nM  60.8 nM  70.2 nM  80.0 nM  88.7 nM  116.2 nM  124.2 nM  125.1 nM  133.5 nM  138.7 nM  144.0 nM  171.1 nM  187.6 nM  191.8 nM  210.1 nM  213.0 nM  214.0 nM  230.8 nM  239.5 nM  259.9 nM  261.5 nM  16.9 nM  38.5 nM  51.7 nM  53.7 nM  54.6 nM  57.8 nM  60.4 nM  65.3 nM  75.7 nM  79.8 nM  90.5 nM  90.7 nM  91.7 nM  92.3 nM  98.0 nM  109.0 nM  121.5 nM  123.1 nM  123.2 nM  125.5 nM  128.8 nM  137.1 nM  142.0 nM  153.3 nM  176.9 nM  181.3 nM  222.5 nM  230.1 nM  249.5 nM  76.33nM  134.95nM  167.77nM  185.80nM  261.12nM  279.51 nM  280.34nM | WB  WB  WB  SB  WB  SB  SB  SB  SB  SB  SB  SB  SB  SB  WB  WB  WB  WB  WB  WB  WB  WB  WB  WB  WB  WB  WB  WB  WB  WB  WB  WB  WB  WB  WB  WB  WB  WB  WB  SB  WB  WB  WB  WB  WB  WB  WB  WB  WB  WB  WB  WB  WB  WB  WB  WB  WB  WB  WB  WB  WB  WB  WB  WB  WB  WB  WB  WB  WB  WB  WB  WB  WB  WB  WB |
| Cp15 | IALDEIHQL  VVFDSTSISL | 1040.4 nM  643.2nM | WB  WB | ALDEIHQLL  IALDEIHQLL  VVFDSTSISL | 11.4 nM  48.9nM  820.1nM | SB  WB  WB |

Estimated binding affinity to H-2K^b^ and HLA-A*0201 using the NetMHCpan 4.0 server. Altogether, 202 8-11mer peptides including 107 H-2K^b^-restricted peptides and 110 HLA-A*0201-restricted peptides were predicted for the *C. parvum* proteins The 15 peptides (marked in yellow bold) were predicted for both H-2K^b^ and HLA-A*0201 binding motifs with high estimated binding affinities. The estimated binding affinities to H-2K^b^ and HLA-A*0201 were measured as IC50 values in nM (aff), and the peptides were selected with theoretical IC50 values ranging from 2.9 to 1179.35 nM, for which the binding level was strong (Rank Threshold for Strong binding peptides, %Rank<0.500) or weak (Rank Threshold for Weak binding peptides, 0.500<%Rank<2.000). Binding affinity values are in nM (aff) and the IC50 value is inversely proportional to the affinity.
