# Supplementary figures and images for: Structural Analyses of a Dominant Cryptosporidium parvum Epitope Presented by H-2Kb Offer New Options To Combat Cryptosporidiosis
Source: mBio. 2023 Jan 5;14(1):e02666-22. doi: 10.1128/mbio.02666-22 (PMC9973275; doi:10.1128/mbio.02666-22)

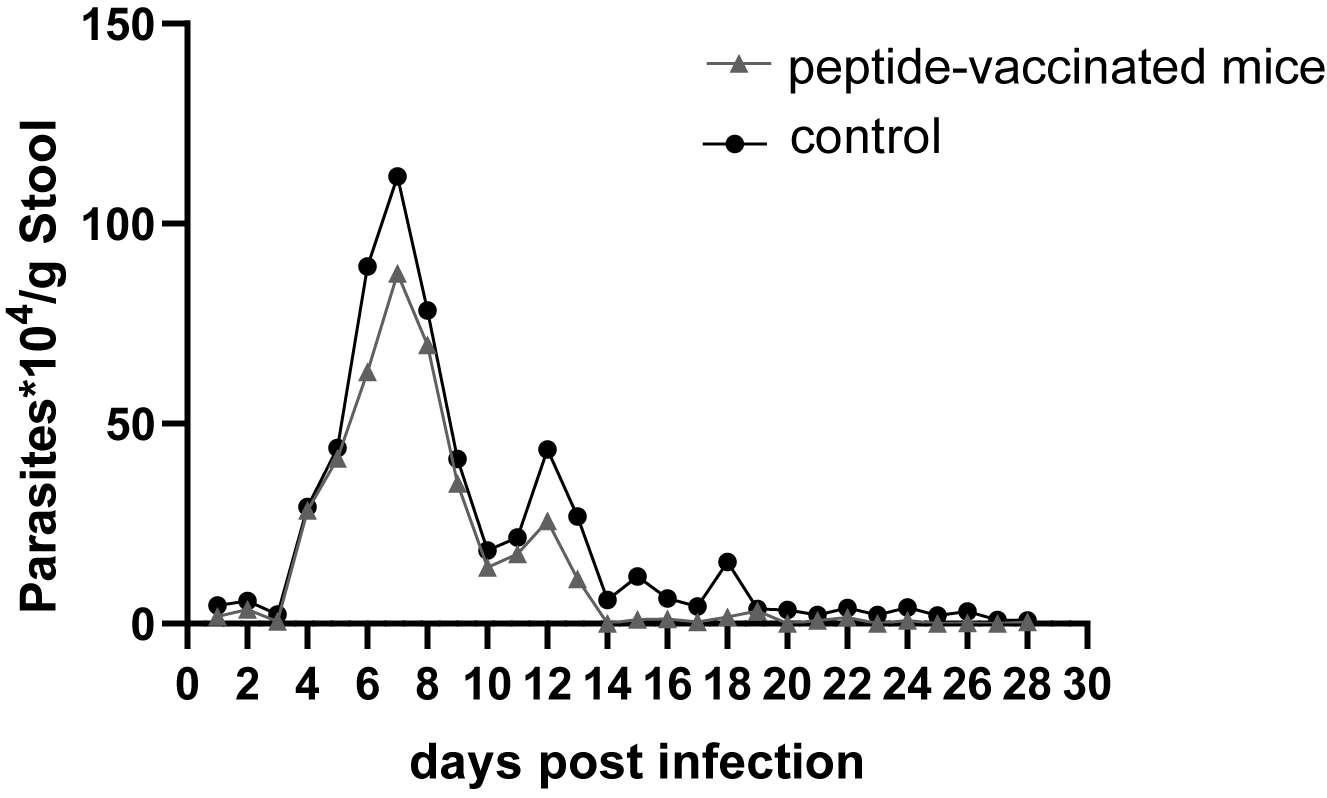

Supplement: FIG S1 [file mbio.02666-22-s0001.tif]

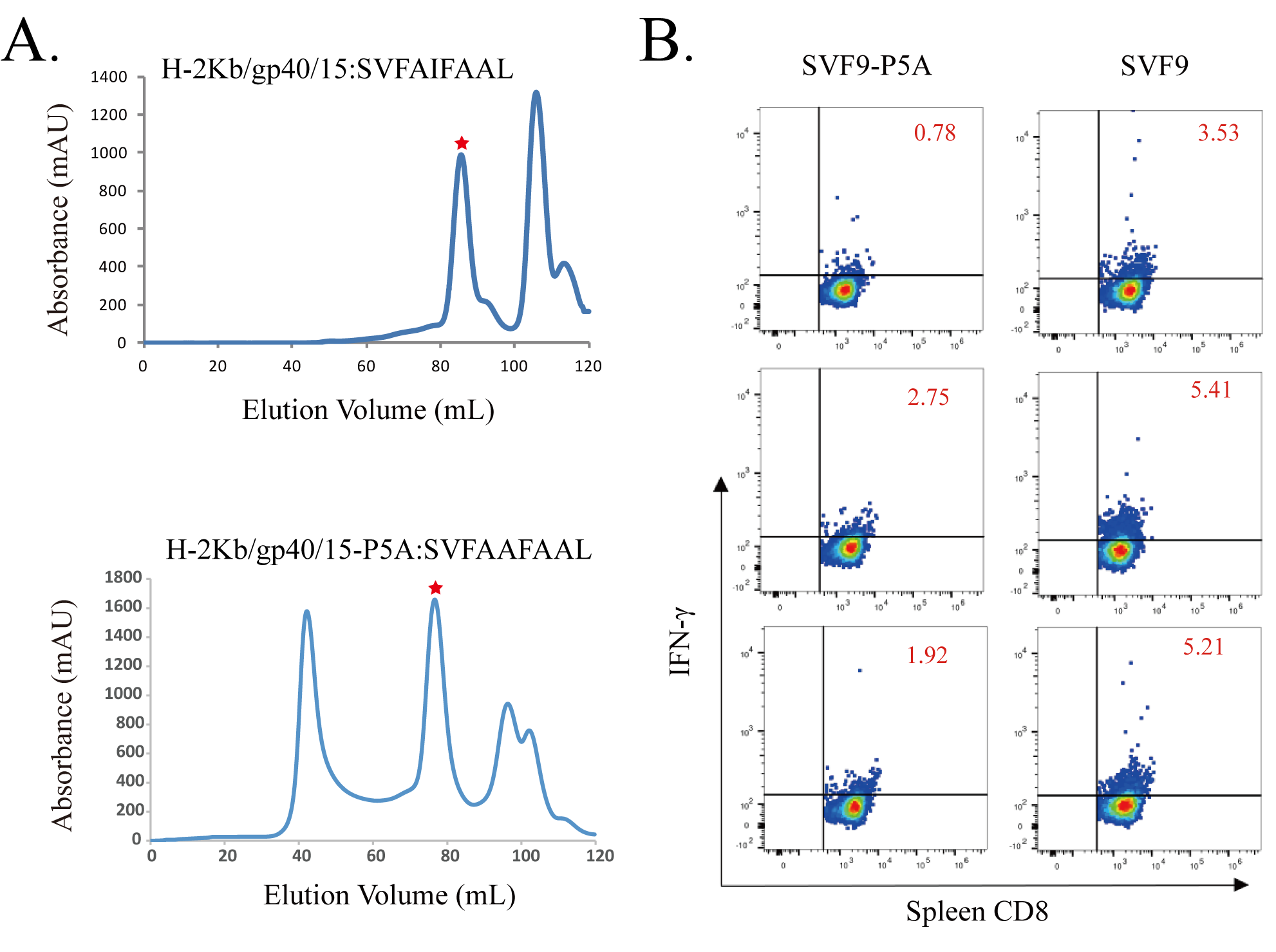

Supplement: FIG S2 [file mbio.02666-22-s0002.tif]

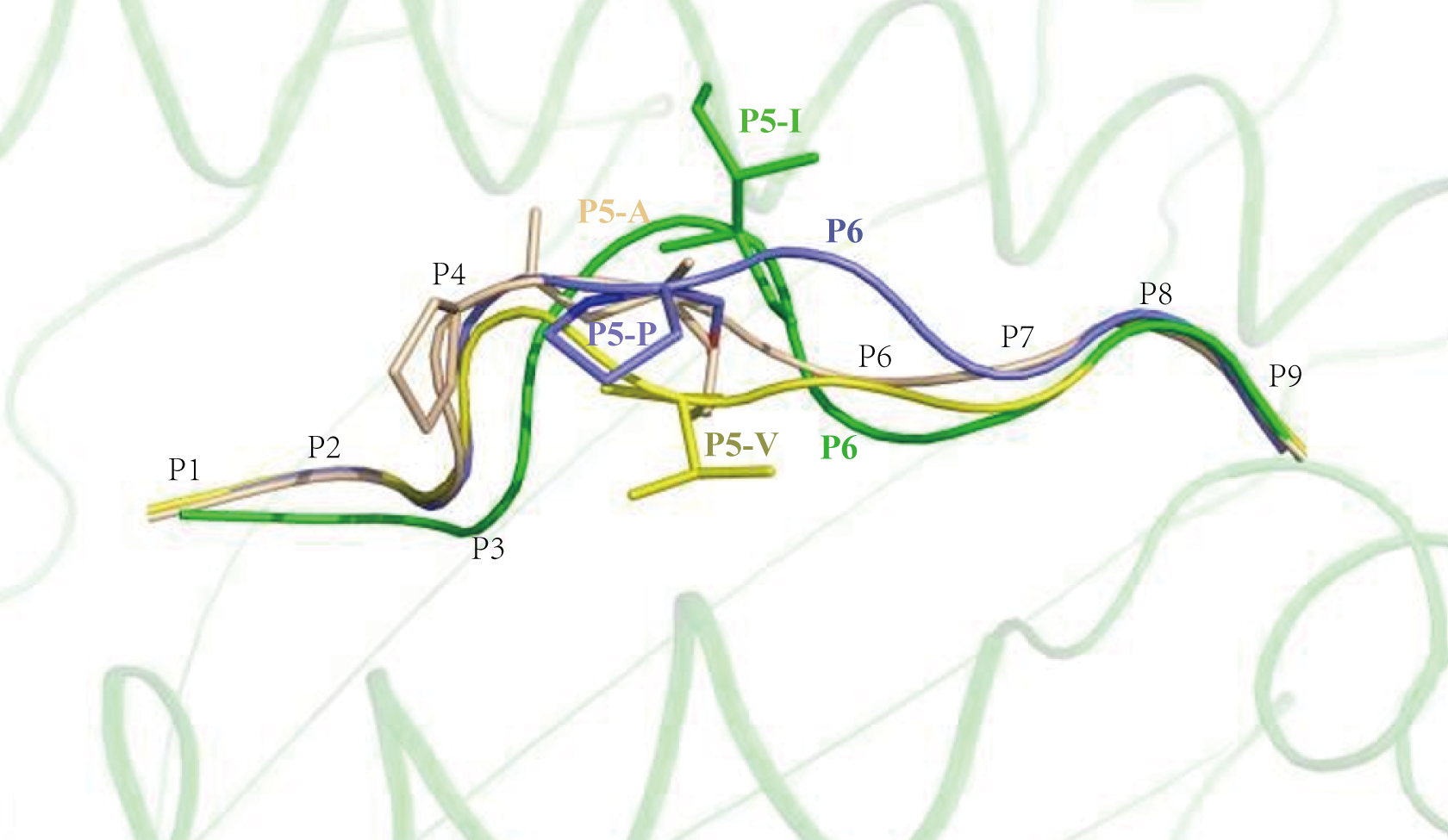

Supplement: FIG S3 [file mbio.02666-22-s0003.tif]
