# Supplementary material for: Structural Analyses of a Dominant Cryptosporidium parvum Epitope Presented by H-2Kb Offer New Options To Combat Cryptosporidiosis
Source: mBio. 2023 Jan 5;14(1):e02666-22. doi: 10.1128/mbio.02666-22 (PMC9973275; doi:10.1128/mbio.02666-22)
Supplement: TABLE S3 [file mbio.02666-22-s0006.doc]

**Table S3. Complete list of nonapeptides selected from *C. parvum*, *C. hominis* and *C. felis* based on the preliminary motif.**

Note: GenBank accession number: Gp40/15, *C. hominis*: ACQ82740.1; *C. parvum*: AAL07532.1; *C. felis: ABH11920.1;* Cp15, *C. hominis*: XP_666956.1; *C. parvum*: AAA28294.1; *C. felis*: KAF74581128.1; Cp23*, C. hominis*: AEJ22864.1; *C. parvum*: AAN31184.1; *C. felis*: KAF7456923.1; HSP70, *C. hominis*: AIT12349.1; *C. parvum*: AGS42174.1; *C. felis*: AKM20838.1; *CSL, C. hominis*: CUV04935.1; *C. parvum*: EAK89290.1; Gp900, *C. hominis*: OLQ8067.1; *C. parvum*: AAC98153.

| Allele | C. parvum | *C. hominis* | | *C.felis* | |
| --- | --- | --- | --- | --- | --- |
| Derived protein | **S**equence^a^ | Sequence^a^ | Sequence Identity (%) | Sequence^a^ | Sequence Identity (%) |
| gp40/15 | SVFAIFAAL  VSAVFSAPA  AIFAALFVL  SAFGLRYIV  SSTSTVAPA  LIIVLLSVI  IIVLLSVIV  RLSLIIVLL | SVFAIFAAL  VSAVFSAPA  AIFAALFVL  SAFGFRYIV  SSSTTVAPA  LIIVLLSVI  IIVLLSVIV  RLSLIIVLL | 100  100  100  88  77  100  100  100 | SVFAIFAAL  VSAVFSAPA  AIFAALFEG  SAFGLRYIV  SSTSTVAPA | 100  100  77  100  100 |
| Cp15 | IALDEIHQL  INTFNVKLI  ALDEIHQLL | IALDEIHQL  INTFNVKLI  ALDEIHQLL | 100  100  100 | ISLDEIHQL  VNVFNVKLI  SLDEIHQLL | 88  77  88 |
| Cp23 | KAVKNPAPI  ELAEKKAQL  KVAENKSAA | KAVKNPAPI  ELAEKKAQL  KVAENKSAA | 100  100  100 | KAVKNPPPI  ELAEKKAQL  KVADNKSAA | 88  100  88 |
| HSP70 | VSRARFEEL  CADYFRATL  KLIERNTTI  SLENYLYNM  IAGLNVMRI  TRIPKVQAL  EGIDYSVAV  NGILNVSAV  DVAPLSLGL  KSTGKSSKI  TIPAKKTQV | VSRARFEEL  CADYFRATL  KLIERNTTI  SLENYLYNM  IAGLNVMRI  TRIPKVQAL  EGIDYSVAV  NGILNVSAV  DVAPLSLGL  KSTGKSSKI  TIPAKKTQV | 100  100  100  100  100  100  100  100  100  100  100 | ISRARFEEL  CADYFRATL  KLIERNTTI  SLENYLYNM  IAGLNVMRI  TRIPKVQAL  EGIDYSVAI  NGILNVSAV  DVAPLSLGL  KSTGKSSKI  TIPAKKTQV | 88  100  100  100  100  100  88  100  100  100  100 |
| CSL | MIWHKSVNL  RITVKYHAL  NSMIWHKSV  ESLSNYNSM  LAFLRLVRK | MIWHKSVDL  RITVKYHAL  NSMIWHKSV  ESLSNYNSM  LAFLRLVRK | 88  100  100  100  100 |  |  |
| Gp900 | MIYDYNSGL  KMLDKYTRM  SVSGVFATV  TSMNWPVSI  SGKYFSGSI  TIPNTYAGV  SAIALVAVI  SANTNFLLV  IMNPLFSLA  AASVIHTAL  VIPSLPSDL  MLFDNSTGV  TGVMIPGSL  AAVGLVAAV  IMNGTIAGI  FAGAYKYAV  LLSQKSAPI  TIAGIVSGI  TGKVIPGSL | MIYDYNSGL  KMLDKYTRM  SVSGVFATV  TSMNWPVSI  SGKYFSGSI  TIPNTYAGV  SAIALVAVI  SADTNFLLV  IMNPLFSLA  AASVIHTAL  VIPSLPSDL  MLFDNSTGV  TGVMIPGSL  AAVGLVAAG  IMNGTIAGI  FAGAYKYAG  LLSQKSAQI  TIAGIVSGI  TGKVIPGSL | 100  100  100  100  100  100  100  88  100  100  100  100  100  88  100  88  88  100  100 |  |  |
