# Supplementary material for: Structural Analyses of a Dominant Cryptosporidium parvum Epitope Presented by H-2Kb Offer New Options To Combat Cryptosporidiosis
Source: mBio. 2023 Jan 5;14(1):e02666-22. doi: 10.1128/mbio.02666-22 (PMC9973275; doi:10.1128/mbio.02666-22)
Supplement: TABLE S4 [file mbio.02666-22-s0007.docx]

**Table S4. Contacts between the public TCR and HLA-A*0201-YLQ and the H-2K^b^ complex.**

| TCR Segment | TCR Residues | HLA-A*0201 Residues | | H-2Kb Residues | |
| --- | --- | --- | --- | --- | --- |
|  |  | Residue | Type Bond | Residue | Type Bond |
| CDR1 α | Arg28 | Glu166 | VDW,HB,SB | Glu166 | VDW,HB,SB |
| CDR1 α | Gln37 | Gln155,Tyr159 | VDW | Arg155, Tyr159 | VDW |
| CDR1 α | Ser38 | Gln155 | VDW,HB | Arg155, | VDW, HB |
| CDR2 α | Tyr57 | Glu154,Gln155, Ala158 | VDW | Glu154,Ala158, | VDW |
| CDR2 α | Ser58 | Glu154,Arg157 | VDW,HB | Glu154, Arg157 | VDW,HB |
| CDR3 α | Asp109 | Arg65,Lys66 | VDW,SB | Arg62,Gln65 | VDW,HB,SB |
| CDR3 α | Arg108 |  |  | Arg62 | VDW |
| CDR1β | Arg38 | Thr73 | VDW |  |  |
| CDR2β | Gln57 | Thr73,Val76 | VDW,SB | Gln72, Ser73,Val76 | VDW,HB |
| CDR2β | Asn58 | Val76 | VDW | Val76 | VDW |
| CDR3β | Asp109 | Ala150,Gln155 | VDW,HB | Glu152,Arg155 | VDW,HB,SB |
| CDR3β | Ile110 | Gln115 | VDW | Arg155 | VDW |
|  | Leu66 |  |  | Gln72 | VDW |
|  | Glu67 |  |  | Gln65 | VDW,HB |

Abbreviations are as follows: FW, framework residue; HB, hydrogen bond (cut-off distance 3.5 Å); SB, salt bridge (cut-off distance 5 Å); VDW, van der Waals (cut-off distance 4 Å).
